# Untargeted metabolome atlas for sleep-related phenotypes in the Hispanic community health study/study of Latinos

**DOI:** 10.1016/j.ebiom.2024.105507

**Published:** 2024-12-17

**Authors:** Ying Zhang, Brian W. Spitzer, Yu Zhang, Danielle A. Wallace, Bing Yu, Qibin Qi, Maria Argos, M Larissa Avilés-Santa, Eric Boerwinkle, Martha L. Daviglus, Robert Kaplan, Jianwen Cai, Susan Redline, Tamar Sofer

**Affiliations:** a Division of Sleep Medicine and Circadian Disorders, Department of Medicine, Brigham and Women's Hospital, Boston, MA, USA; b CardioVascular Institute, Beth Israel Deaconess Medical Center, Boston, MA, USA; c Department of Medicine, Harvard Medical School, Boston, MA, USA; d Department of Epidemiology, School of Public Health, The University of Texas Health Science Center at Houston, Houston, TX, USA; e Department of Epidemiology and Population Health, Albert Einstein College of Medicine, Bronx, NY, USA; f Department of Epidemiology and Biostatistics, School of Public Health, University of Illinois Chicago, Chicago, IL, USA; g Department of Environmental Health, School of Public Health, Boston University, Boston, MA, USA; h Division of Clinical and Health Services Research, National Institute on Minority Health and Health Disparities, National Institutes of Health, Bethesda, MD, USA; i Institute for Minority Health Research, University of Illinois at Chicago, Chicago, IL, USA; j Division of Public Health Sciences, Fred Hutchinson Cancer Center, Seattle, WA, USA; k Collaborative Studies Coordinating Center, Department of Biostatistics, University of North Carolina at Chapel Hill, Chapel Hill, NC, 27599, USA; l Department of Biostatistics, Harvard T.H. Chan School of Public Health, Boston, MA, 02115, USA

**Keywords:** Metabolomics, Sleep, Atlas, Bipartite network, Hispanic or latino

## Abstract

**Background:**

Sleep is essential to maintaining health and wellbeing of individuals, influencing a variety of outcomes from mental health to cardiometabolic disease. This study aims to assess the relationships between various sleep-related phenotypes and blood metabolites.

**Methods:**

Utilising data from the Hispanic Community Health Study/Study of Latinos, we performed association analyses between 40 sleep-related phenotypes, grouped in several domains (sleep disordered breathing (SDB), sleep duration, sleep timing, self-reported insomnia symptoms, excessive daytime sleepiness (EDS), and heart rate during sleep), and 768 metabolites measured via untargeted metabolomics profiling. Network analysis was employed to visualise and interpret the associations between sleep phenotypes and metabolites.

**Findings:**

The patterns of statistically significant associations between sleep phenotypes and metabolites differed by superpathways, and highlighted subpathways of interest for future studies. For example, primary bile acid metabolism showed the highest cumulative percentage of statistically significant associations across all sleep phenotype domains except for SDB and EDS phenotypes. Several metabolites were associated with multiple sleep phenotypes, from a few domains. Glycochenodeoxycholate, vanillyl mandelate (VMA) and 1-stearoyl-2-oleoyl-GPE (18:0/18:1) were associated with the highest number of sleep phenotypes, while pregnenolone sulfate was associated with all sleep phenotype domains except for sleep duration. N-lactoyl amino acids such as N-lactoyl phenylalanine (lac-Phe), were associated with sleep duration, SDB, sleep timing and heart rate during sleep.

**Interpretation:**

This atlas of sleep–metabolite associations will facilitate hypothesis generation and further study of the metabolic underpinnings of sleep health.

**Funding:**

R01HL161012, R35HL135818, R01AG80598.

**Research in context**

**Evidence before this study**

The authors searched standard databases such as PubMed and Google Scholar to identify studies reporting metabolomics associations with sleep-related phenotypes. Studies linking metabolites to sleep-related phenotypes are increasingly reported.

**Added value of this study**

This study used a relatively large dataset, in terms of both the sample size (n ≈ 6000) and the number of metabolites (over 700) to estimate the associations between metabolites and a range of sleep-related phenotypes from multiple domains. The authors used the resulting atlas to identify patterns of associations across sleep-related phenotype domains, and metabolite pathways. Metabolites that were linked to multiple sleep phenotypes were studied.

**Implications of all the available evidence**

The patterns of statistically significant associations between sleep phenotypes and metabolites differed by superpathways, highlighting subpathways of interest for future studies. Several metabolites, such as glycochenodeoxycholate, vanillyl mandelate (VMA) and 1-stearoyl-2-oleoyl-GPE (18:0/18:1), and N-lactoyl amino acids, were associated with multiple sleep phenotypes, from a few domains. The authors expect that the generated atlas of associations will be used to develop in-depth studies of sleep and circadian measures and contextualise them within other aspects of health and disease.

## Introduction

Sleep plays an important role in the health and wellbeing of individuals. Insufficient quality, timing, and duration of sleep have a major public health impact, and are associated with daytime sleepiness, poor mental health, impaired cognitive function, and increased risk of cardiovascular morbidity and mortality.^1^, ^2^, ^3^ Sleep is increasingly recognised as a crucial factor in cardiovascular health, evident by the addition of sleep duration to the “life's essential 8” metric developed by the American Heart Association.^4^ In addition to sleep duration, measures of suboptimal sleep, such sleep disturbances and quality (or insomnia symptoms), irregularity of sleep timing, sleep fragmentation, and sleep disordered breathing (SDB), are also associated with poor health outcomes.^5^ In fact, there is growing recognition of the importance in measuring and characterising multi-dimensional sleep health—a framework that concurrently considers these varied aspects of sleep.^6^, ^7^, ^8^

Despite the strong epidemiological evidence observed in many cohort and clinical studies for the connection between suboptimal sleep health and increased risks for poor health outcomes, the biology and physiology behind these links are not fully understood. While many sleep behaviours and outcomes share some underlying genetic and physiological pathways,^9^, ^10^, ^11^ or have, potentially bidirectional, causal relationships,^12^ there may also be distinct mechanisms that underlie specific sleep disturbances or sleep subtypes.^13^, ^14^, ^15^ Untangling these shared and distinct mechanisms underlying sleep phenotypes has the potential to inform sleep health intervention efforts.

Biological sampling to measure molecular markers of health, such as in metabolomics, can be used to investigate the mechanisms and pathways underpinning sleep phenotypes. Metabolites are small molecules produced in the formation and/or breakdown of endogenous or exogenous substances and are oriented at the closest layer to phenotypes compared to other underlying biochemical layers (e.g., genome, transcriptome and proteome). The increasing availability of large datasets with untargeted metabolomics profiling has unveiled metabolic outcomes and correlates of numerous health phenotypes, including sleep measures.^16^, ^17^, ^18^, ^19^, ^20^, ^21^, ^22^, ^23^, ^24^, ^25^, ^26^, ^27^, ^28^, ^29^, ^30^, ^31^ A large-scale study of metabolites in relation to sleep phenotypes may shed light on how underlying biological processes may converge and differ among common sleep phenotypes, the complex interplay between sleep and the metabolic environment, and, ultimately, potential interactions among sleep disorders and progression of cardiometabolic and other health conditions. Here, we study the associations between a range of sleep phenotypes and the metabolic environment in a large population-based study using a high-dimensional set of measured metabolites. We create an “atlas”—a resource for the sleep research community that will facilitate hypotheses formulation and accelerate studies on sleep and its association with other health outcomes.

**Fig. 1:**
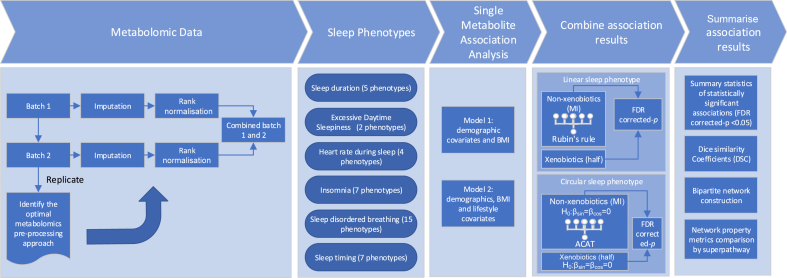
**Study design diagram**. MI: multiple imputation; half: imputation of the missing values for each metabolite with half of the lowest non-missing value of that metabolite across the sample within the batch; ACAT: the aggregated Cauchy association test; FDR: false discovery rate.

Our study has taken a comprehensive approach (Fig. 1), covering key sleep-related phenotypes including sleep disordered breathing (SDB), sleep duration, sleep timing, self-reported insomnia symptoms, excessive daytime sleepiness (EDS), and heart rate (HR) during sleep. Each domain of sleep-related phenotypes provides a different perspective on sleep, while together may highlight some shared biological processes within this complex physiological phenomenon. We also conducted network analysis to better understand the interconnectedness between multiple sleep phenotypes and metabolites –representing significant associations as links in a bipartite network allows for simultaneous visualisation of many associations, enabling researchers to observe connectivity patterns that might otherwise be obscured when looking at the individual relationships. By reporting a large number of associations between metabolites and sleep phenotypes, this resource may provide researchers with a starting point for more targeted inquiries into the metabolic environment changes induced by sleep disorders, and facilitate hypothesis generation for future metabolomic sleep research. This may ultimately contribute to our understanding of the pathogenesis of sleep disorders and pave the way for developing more effective diagnostic and therapeutic strategies.

## Methods

### The Hispanic community health study/study of Latinos

The Hispanic Community Health Study/Study of Latinos (HCHS/SOL) is a prospective community-based cohort study of 16,415 Hispanic/Latino individuals aged 18–74 years at the baseline examination (2008–2011). Study participants were selected using a multi-stage stratified random sampling from four geographic regions: Bronx NY, Chicago IL, Miami FL, and San Diego CA.^32^^,^^33^ Sex was ascribed by the interviewer as male or female, and was not verified by other means. Description of major ancillary studies and findings in the context of cardiovascular health is provided in a prior publication.^34^ Fasting blood samples were collected at the baseline examination, and within the subsequent week, 14,440 of these participants underwent an evaluation for SDB using a validated Type 3 home sleep apnoea test (ARES Unicorder 5.2; B-Alert, Carlsbad, CA) that measured nasal airflow, position, snoring, heart rate and oxyhaemoglobin saturation with measures of SDB scored by a central reading centre as detailed previously.^35^ All sleep phenotypes used, and their definitions are provided in Supplementary Note 1.

### Metabolomics profiling

From those who attended the HCHS/SOL baseline assessment and also underwent genotyping (12,803 of the study individuals^36^), 4002 individuals were selected at random for metabolomics profiling of fasting serum samples collected at baseline (metabolomics batch 1, processed in 2017). In 2021, additional 2368 serum samples from 2330 participants, also collected at baseline, were profiled in a second metabolomics batch 2. This batch included repeated measures within the batch for quality control purposes. Serum samples were stored at −70 °C at the HCHS/SOL Core Laboratory at the University of Minnesota until analysis by Metabolon, Inc (Durham, NC) in 2017 (batch 1) and 2021 (batch 2). Serum samples were then extracted and prepared using Metabolon's standard solvent extraction method. Extracts were split into five fractions to use in four liquid chromatography-mass spectrometry (LC-MS)-based metabolomic quantification platforms (two reverse phase methods with positive ion mode electrospray ionisation (EI), one reverse phase method with negative ion mode EI, and one hydrophilic interaction liquid chromatography with negative ion mode EI), with the fifth fraction reserved for backup. Instrument variability was assessed by calculating the median relative standard deviation (SD) for the internal standards added to each sample prior to injection into the mass spectrometers. Overall process variability was determined by calculating the median relative SD for all endogenous metabolites (i.e., non-instrument standards) present in 100% of the technical replicate samples. Following a recent publication,^37^ we re-labelled a few metabolites, previously labelled as 1-carboxyethyl molecules, to N-lactoyl molecules.

### Metabolomic data pre-processing

Preprocessing of the metabolomic data is described in Supplementary Figure S1. First, we removed batch 2 individuals who overlapped with batch 1 and replicate samples from the same individuals, resulting in 2178 remaining batch 2 observations. We then computed percentages of missing values of each metabolite in each batch separately. We excluded metabolites with missing values in more than 75% of the individuals in either batch. For xenobiotic metabolites (metabolite annotation was provide by Metabolon), we assumed that missing values were due to concentrations below the minimum detection limits, thus imputed the missing values for each metabolite with half of the lowest non-missing value of that metabolite across the sample within the batch. For non-xenobiotic metabolites, we applied multiple imputation using the futuremice function from the R mice package (version 3.15.0) that implements fully conditional imputation in a computationally efficient manner (using parallelisation). Each variable in the imputation dataset is imputed (if it has missing values) using a model that predicts potential values based on other variables in the dataset. The dataset was imputed 5 times to generate 5 completed datasets. Differences between the computed datasets are due to randomness (e.g., random residuals added to the predicted values of a variable). We imputed metabolites, separately in each batch, together with a set of covariates that are strongly linked to the metabolic environment: age, sex, BMI, waist-to-hip ratio, fasting insulin, fasting glucose, type 2 diabetes status (American Diabetes Association definition: fasting glucose≥126 mg/dL, or post-OGTT glucose≥200 mg/dL or A1C ≥ 6.5%, or self-report of diabetes), estimated glomerular filtration rate, and lipid measures—total cholesterol, triglycerides, high- and low-density lipoprotein measures. We then rank normalised the 768 metabolite measures passing quality control in each batch separately and each imputed dataset separately, and finally, aggregated data from the two batches, such that the “first” imputed batch 1 dataset was aggregated with the “first” imputed batch 2 dataset, and so on. Supplementary Note 2 describes the analysis we performed to inform the metabolite imputation strategy.

### Sleep phenotypes and modelling approach

We used 40 sleep phenotypes from 6 domains, described in detail in Supplementary Note 1. In brief, these included: self-reported sleep duration (average sleep duration on weekdays, weekend days, and over all days, short and long sleep on weekdays), heart rate during sleep (minimum, maximum, average, and standard deviation of HR), self-reported insomnia symptoms (the women health initiative insomnia rating scale (WHIIRS), its component questions, and self-reported sleeping pill use), daytime and excessive daytime sleepiness (EDS), measured via the Epworth sleepiness scale, sleep disordered breathing (SDB, including measures of breathing disturbances during sleep, mostly measured using a home sleep apnoea testing device: respiratory event indices (REIs), measures of oxyhaemoglobin saturation during sleep, measures of respiratory event lengths; and self-reported snoring, because SDB and snoring often co-occur and share causal determinants such as upper airway collapsibility), and sleep timing (bed time and wake time as well as sleep midpoint during weekend and weekend days, and social jetlag—the difference in sleep midpoint between weekend and weekdays). In figures and tables, these domains are referred to as Duration, HR, Insomnia, EDS, SDB, and Timing. Phenotypes in the sleep timing category were analysed as “circular” variables, i.e., to avoid bias due to day thresholding (i.e., defining a day by midnight) and to account for the fact that 11:59 PM (23:59) is adjacent to 12:01 AM (00:01). Other sleep phenotypes were treated as linear or binary (when dichotomised). Because EDS is a cardinal symptom of severe SDB, but may be caused by other factors such as insufficient sleep duration and quality independent of SDB, we used a data-driven approach to select a domain for the EDS phenotypes. As described later, we computed the correlations between metabolite associations of each of the non-circular sleep-related phenotypes (i.e., the correlation between the estimated association effect sizes between the phenotype and all considered metabolites), and visualised the results via a heatmap. EDS as a separate domain was selected based on this visualisation.

Two nested conceptual models were used in the analyses. Model 1 adjusted for batch number, demographic, and baseline clinical variables, including age, sex, field centre, self-reported Hispanic/Latino background (Mexican, Puerto Rican, Cuban, Central American, Dominican, South American, and other/multi) and body mass index (BMI). Model 2 further adjusted for lifestyle variables—alcohol use (never, former, current), cigarette use (never, former, current), total physical activity (MET-min/day, computed based on self-reported time spent doing physical activities), and diet (Alternative Healthy Eating Index 2010, computed based on 24 h dietary recall data) in addition to Model 1 covariates. To appropriately model covariates, we evaluated potential nonlinearity of continuous confounders, including age, BMI, physical activity, and diet, in relation to metabolite levels. For each metabolite, we used all individuals with available metabolite levels (i.e., excluded individuals with missing values), and fit five association models, each adjusted for Model 1 covariates only and the continuous covariate of interest (if not already included in model 1). These association models considered 5 possible modelling of the continuous covariate of interest: linear, and four cubic spline models (with 1, 2, 3, and 4 knots). We computed the Akaike Information Criterion (AIC) for each model, and the model with the lowest AIC value was selected for the main analysis of this metabolite and covariate. Within this model selection rule, we prioritised a simpler model compared to a more complex model using the standard rule of thumb where if the AIC of a complex model was lower by less than 2 points compared to the simpler model, we chose the simpler one.

Sleep phenotypes were used as exposures with metabolites as outcomes. When testing the association of a circular phenotype (i.e., sleep timing) with a metabolite, we first converted the sleep timing phenotype into radians (with 12am serving as the 0) and then computed the sine and cosine of these radians. We used the sine and cosine terms as predictors in the regression,^38^ and tested their association with the metabolite using the multivariate Wald test, accounting for two predictors and their estimated covariance. All association analyses were performed using the *survey* R package (version 4.1) to account for the HCHS/SOL sampling design and provide effect estimates relevant for the HCHS/SOL target population.

### Estimating associations between metabolites and sleep phenotypes

We separately assessed the association between each metabolite's concentration level, as an outcome, and each sleep phenotype, as a predictor, using Model 1 and Model 2 covariates as described above, in a single metabolite association analysis. As described in Fig. 1, xenobiotic metabolites were imputed once, while missing values in other metabolites values were imputed 5 times. Thus, we estimated the associations of non-xenobiotic metabolites with sleep phenotypes 5 times (using each of the completed datasets), and then combined the resulting estimated associations. Here we treated the linearly-modelled sleep phenotypes differently from the circular ones. The estimated metabolite associations with linearly-modelled sleep phenotypes were combined using Rubin's rule.^39^ Sleep timing phenotypes cannot be combined in the same manner, because there is no method to combine the covariance between the sine and cosine terms across several completed datasets. Instead, we focused on testing and aggregated the *p*-value of the multivariate Wald test using the aggregated Cauchy association test (ACAT^40^). The ACAT test was developed in the context of genetic association analyses, but it is appropriate for our settings, because it allows for the aggregated tests to be based on correlated data. Finally, after combining association results so we had one *p*-value per sleep phenotype-metabolite association (per model), we implemented the Benjamini-Hochberg method to control the false discovery rate (FDR) for multiple testing across all metabolites within each model and sex stratum for each sleep phenotype.^41^ Any association that resulted in an FDR-corrected *p* < 0.05 in Model 1 was considered statistically significant. To assess whether some associations are potentially driven by confounding effects from other lifestyle measures, we examined if Model 2 effect estimates fall within the 95% CI of Model 1 effect estimates from each regression model for non-timing sleep phenotypes (as the latter phenotypes do not have effect estimates). In a secondary analysis, we also performed sex-stratified analyses using the same analytic approach. For descriptive purposes, we computed the number of sleep traits that each metabolite was associated with at the FDR-corrected *p* < 0.05 Model 1 threshold, and identified the top 10% metabolites based on number of sleep trait associations. A Spearman correlation matrix was computed using the squared effect estimates for each pair of metabolites and sleep phenotypes for sex-combined and sex-stratified analysis.

### Assessment of potential metabolite biomarkers for sleep-related phenotypes

We evaluated whether there is any specific metabolite that could potentially be used as a biomarker for one of the phenotypes. Based on the metabolite-sleep phenotype results, we selected associations with *p*-value <10^−5^. Among those, for each phenotype that had more than one metabolite meeting this criterion, we picked the top metabolite with respect to its *p*-value. Finally, we assessed how well the metabolite explains the corresponding sleep-related phenotypes, using adjusted R squared. We fit linear or logistic regression models (survey weighted) with the sleep phenotype as the outcome, and the metabolite as an exposure. One model used only the metabolite, and did not adjust for anything else. This model assessed the potential of using the metabolite individually as a biomarker. Another model used age, sex, study centre, Hispanic background, and BMI as covariates (i.e., Model 1 covariates), and did not include the metabolite. A third model adjusted for Model 1 covariates and included the metabolite. This model assessed the improvement in variance explained, over a basic model, due to the metabolite. We computed the adjusted R squared in the three models.

### Sensitivity analysis for the aggregation of two metabolomics batches

Compared to our primary approach of aggregating the two metabolomics batches, we considered whether a more appropriate approach would be to perform sleep–metabolite association analysis in each batch separately, and then combine the results via a meta-analysis. For this comparison, we used 7 sleep-related phenotypes: hypoxia, long sleep during weekdays, WHIIRS, average sleep duration, REI3%, minimum SpO2 during sleep, and mean HR during sleep. We removed individuals from batch 2 who live in the same household as individuals in batch 1, to prevent inappropriate inference of standard errors (batch combined analysis accounts for this structure using robust standard errors). In both analyses (batch aggregated and separated), metabolites were rank-normalised in each batch separately. Next, for each metabolite, we performed linear regression analysis using individuals with observed values for this metabolite, during both the primary (batch combined) and sensitivity analysis (separate batch followed by meta-analysis) approaches. For this comparison we used linear regression, and not survey-based analysis, based survey-based analysis is not recommended when there are less than 2000 individuals in a dataset, as was true for batch 2. Finally, for each compared phenotype, we computed the Pearson and Spearman correlations between the estimated association effect sizes across all metabolites to determine whether the results are different or similar between the two modelling approaches.

### Similarity of associations between sleep-related phenotypes and domains

We summarised the associations between sleep phenotypes, individually and categorised by domains, and metabolites, individually and categorised by pathways, where superpathways and subpathways were provided by Metabolon in a metabolite annotation file. Similarities of these associations between a pair of phenotypes or domains were estimated with the Dice Similarity Coefficient (DSC)^42^ defined as

$$DSC=\frac{2\left|X\cap Y\right|}{\left|X\right|\;+\;\left|Y\right|}$$

Where *X* and *Y* represent the set of metabolites with statistically significant associations (FDR-corrected *p* < 0.05) with two sleep phenotypes or domains. DSC takes values between 0 and 1, where 0 indicates no similarity while 1 indicates full overlap between the two sets.

### Bipartite network analysis

Bipartite network is a type of network in which there are two groups of nodes (here: sleep phenotypes and metabolites), and links, or edges, can only exist between the two types of nodes but not within a group of nodes. We constructed a bipartite network using the sleep phenotypes and metabolites, where an edge was added between a sleep phenotype-metabolite pair if their association was statistically significant (FDR-corrected *p* < 0.05) in Model 1 association analysis. The network is built based on an incidence matrix, with rows corresponding to sleep phenotypes, columns represent metabolites, and the i,j cell in this matrix has a value of 1 if the i th sleep phenotype has a statistically significant association with the j th metabolite, and 0 otherwise. We then aggregated metabolites by sub- and super-pathway, and sleep phenotypes by domain, which results in a consolidated incidence matrix in which a value of 1 in cell (*m,n)* indicating any statistically significant association between the *m*th sleep phenotype domain and the *n*th metabolite sub- and super-pathway and a weight matrix documenting the number of total statistically significant associations between the *m*th sleep phenotype domain and the *n*th metabolite sub- and super-pathway. Several network property metrics were computed to offer insights into the structure and associations of the sleep domain—metabolite sub- and super-pathway network.

For visualisation, we converted the bipartite network into a univariate network and visualised the network using the Fruchterman-Reingold force-directed algorithm, which optimises the placement of nodes based on connectivity similarity between nodes, where similar connectivity is reflected as proximity of nodes,^43^ providing an intuitive spatial representation of the network structure.

### Statistics

Associations of metabolite levels with sleep-related phenotypes were based on the Wald test for non-circular variables, i.e., when a single effect estimate was available for a phenotype. The Wald tests was computed after effect estimates and their SEs were combined across multiply-imputed datasets using Rubin's rule. For circular variables for which two coefficients were estimated corresponding to sine and cosine terms, we first applied the multivariate Wald test, and next combined *p*-values across the multiply-imputed datasets using the ACAT method, because there is no existing method to combine effect estimates and covariance matrices from multiple correlated variables in the multiple imputation settings.

All analyses were done in R version 4.2.3. The function svyglm from the *survey* package was used for survey-weighted generalised linear regression models. Cubic splines for modelling of continuous covariates were generated using the ns function from the *splines* package. The *car* package (version 3.1) was used for multivariate Wald test. Adjusted R square was computed using the rsq function from the *rsq* package. The *bipartite* package (version 2.18) was used for bipartite network analysis, and *igraph* (version 1.5) and *ggnetwork* (version 0.5) packages were used for visualising network graph.

### Ethics

The HCHS/SOL was approved by the institutional review boards (IRBs) at each field centre, where all participants gave written informed consent, and by the Non-Biomedical IRB at the University of North Carolina at Chapel Hill, to the HCHS/SOL Data Coordinating Center. All IRBs approving the HCHS/SOL study are: Non-Biomedical IRB at the University of North Carolina at Chapel Hill. Chapel Hill, NC (study #07-1003); Einstein IRB at the Albert Einstein College of Medicine of Yeshiva University. Bronx, NY (reference #007788); IRB at Office for the Protection of Research Subjects (OPRS), University of Illinois at Chicago. Chicago, IL (protocol #2013-1261); Human Subject Research Office, University of Miami. Miami, FL (study number 20070461); Institutional Review Board of San Diego State University, San Diego, CA (protocol number 1586091). All methods and analyses of HCHS/SOL participants’ materials and data were carried out in accordance with human subject research guidelines and regulations. This work was approved by the Mass General Brigham IRB (protocol #2022P001237) and by the Beth Israel Deaconess Medical Center Committee on Clinical Investigations (protocol #2023P000277).

### Role of funders

As a study that used previously collected data, the funding source did not have any role in the study design, analysis, interpretation, or decision to submit this paper for publication.

## Results

### Study sample characteristics

Table 1 characterises the HCHS/SOL target population (using means and percentages weighted to account for study participation), with more comprehensive data, including summary measures of all sleep-related phenotypes, and of common medications, provided in Supplementary Table S1. The analytic sample, batch 1 and batch 2 metabolomic dataset combined, included 6180 participants with a mean age of 44.31 years (SD = 15.27), of whom 40.1% were males, 20.7% did not report alcohol use, 58.5% never smoked, and 11.7% had moderate-to-severe OSA (REI3%≥15). The average sleep duration was 7.98 h (SD = 1.46), while 22.7% reported restless or very restless sleep on a typical night in the last month, and 15.8% reported excessive sleepiness (ESS>10). Batch 2 participants were older compared to batch 1—the mean age is 41.54 years (SD) in batch 1 and 51.11 years (SD) in batch 2. The baseline rates of diabetes and hypertension were higher in batch 2 (diabetes: 29.4%; hypertensions: 44.1%) compared to batch 1 (diabetes: 20.1%; hypertensions: 31.5%), consistent with the age difference of participants between the two batches.

**Table 1:** HCHS/SOL target population characteristics by sex, batch, and overall.

Mean (SD)^a^	Female	Male	Batch 1	Batch 2	Overall
N	3704	2476	4002	2178	6180
Age	44.98 (15.17)	43.56 (15.35)	41.54 (15.18)	51.11 (13.21)	44.31 (15.27)
Sex = Male (%)	0 (0.0)	2476 (100.0)	1705 (42.6)	771 (35.4)	2476 (40.1)
BMI	30.15 (6.69)	28.78 (5.37)	29.42 (6.27)	29.72 (5.80)	29.50 (6.14)
Current alcohol drinking (%)	1401 (37.9)	1490 (60.2)	1973 (49.3)	918 (42.3)	2891 (46.8)
Current smoking (%)	596 (16.1)	668 (27.1)	863 (21.6)	401 (18.5)	1264 (20.5)
Physical activity (MET-min/day)	424.88 (714.71)	879.70 (1174.88)	697.66 (1020.41)	496.61 (880.66)	639.60 (986.25)
The Alternate Healthy Eating Index (2010)	46.79 (7.42)	48.74 (7.47)	47.28 (7.52)	48.75 (7.36)	47.71 (7.50)
OSA status = OSA (%)^b^	260 (7.9)	383 (17.4)	388 (10.9)	255 (13.0)	643 (11.7)
Baseline Diabetes status (ADA) = Yes (%)^c^	863 (23.3)	579 (23.4)	803 (20.1)	639 (29.4)	1442 (23.3)
Baseline Hypertension status = Yes (%)^d^	1357 (36.6)	865 (34.9)	1261 (31.5)	961 (44.1)	2222 (36.0)
Sleep Duration (hours)	8.04 (1.49)	7.90 (1.42)	7.97 (1.45)	7.98 (1.49)	7.98 (1.46)
Women's Health Initiative Insomnia Rating Scale (WHIIRS) total score	7.64 (5.58)	6.16 (5.13)	6.78 (5.37)	7.34 (5.54)	6.95 (5.42)
Epworth Sleepiness Scale (ESS) total score	5.44 (4.71)	5.87 (4.87)	5.62 (4.71)	5.70 (4.99)	5.64 (4.79)

a Means and percentages have been weighted to provide values representative of the HCHS/SOL target population.

b OSA was defined as respiratory event index ≥15, with events counted based on 3% oxyhaemoglobin desaturation.

c Baseline diabetes are based on American Diabetes Association definition (Diabetes Care 2010; 33:S62-69), defined as fasting glucose ≥126 mg/dL, or post-OGTT glucose ≥200 mg/dL or A1C ≥ 6.5%, or self-report of diabetes.

d Baseline hypertension is defined as systolic or diastolic blood pressure greater than or equal to 140/90 respectively, or current use of antihypertensive medications.

### Modelling of continuous covariates

Supplementary Table S2 compares AIC from models treating continuous covariates (age, BMI, diet, and physical activity) as linear versus nonlinear terms (i.e., cubic splines with different knot numbers) and reported the selected optimal modelling for each continuous covariate. For age, only 27% of metabolites had a linear association, while 45%, 68%, and 69% of metabolites had linear associations with BMI, physical activity, and diet measures, respectively. The selected linear or spline models were used in subsequent analyses of the corresponding metabolite.

### Results from sleep phenotype-metabolite association analysis

The single metabolite association analysis was conducted in pair-wise fashion between 40 sleep phenotypes from six domains (i.e., sleep duration, HR, insomnia, EDS, SDB, and sleep timing) and 768 metabolites, including 113 unknown metabolites and 77 xenobiotic metabolites.

34 sleep phenotypes had statistically significant associations with at least one metabolite (Table 2). When limited to Model 1, the median number of significant associations for each sleep phenotype is 14 (range: 0–307), corresponding to 1.82% (range: 0%–39.97%) of all tested metabolites. The number of statistically significant associations is much lower among dichotomised sleep phenotypes (median: 7; range: 0–74) compared to non-dichotomised sleep phenotypes (median: 27; range: 0–307), likely corresponding to loss of power due to dichotomisation. Phenotypes representing heart rate during sleep domain had the highest number of statistically significant associations with metabolites (median: 177, range: 2–307), followed by the sleep timing domain (median: 47, range: 0–135). The SDB domain had the lowest number of statistically significant associations with tested metabolites (median: 8, range: 0–60) (Supplementary Table S3).

**Table 2:** Statistically significant metabolite associations by sleep phenotype.

Sleep phenotype	Number of statistically significant associations	Percentage of statistically significant associations (%)	Sleep phenotype domain	Sleep phenotype dichotomised or continuous
Weekday long sleep	2	0.26	Duration	Dichotomised
Weekday short sleep	0	0	Duration	Dichotomised
Sleep duration	63	8.2	Duration	Continuous
Weekday sleep duration	68	8.85	Duration	Continuous
Weekend sleep duration	0	0	Duration	Continuous
ESS	40	5.21	EDS	Continuous
ESS>10	9	1.17	EDS	Dichotomised
Min HR during sleep	219	28.52	HR	Continuous
Max HR during sleep	135	17.58	HR	Continuous
Avg HR during sleep	307	39.97	HR	Continuous
Std HR during sleep	2	0.26	HR	Continuous
Difficulty back to sleep	0	0	Insomnia	Dichotomised
Early wake	14	1.82	Insomnia	Dichotomised
Difficulty fall asleep	14	1.82	Insomnia	Dichotomised
Frequent wake	0	0	Insomnia	Dichotomised
Sleep pill	74	9.64	Insomnia	Dichotomised
Restless sleep	7	0.91	Insomnia	Dichotomised
WHIIRS	25	3.26	Insomnia	Continuous
Avg event duration	0	0	SDB	Continuous
Hypoxic burden	6	0.78	SDB	Continuous
per90	3	0.39	SDB	Dichotomised
rei0	15	1.95	SDB	Continuous
rei0 > 15	3	0.39	SDB	Dichotomised
rei0 > 5	7	0.91	SDB	Dichotomised
Total event count	12	1.56	SDB	Continuous
Total event duration	9	1.17	SDB	Continuous
rei3	60	7.81	SDB	Continuous
rei3 > 15	11	1.43	SDB	Dichotomised
rei3 > 5	32	4.17	SDB	Dichotomised
Min o2	27	3.52	SDB	Continuous
Avg o2	25	3.26	SDB	Continuous
Perlt90	2	0.26	SDB	Continuous
Snore	4	0.52	SDB	Dichotomised
Social jetlag	0	0	Timing	Continuous
Weekday bed time	132	17.19	Timing	Continuous
Weekday midpoint time	135	17.58	Timing	Continuous
Weekday wake time	98	12.76	Timing	Continuous
Weekend bed time	46	5.99	Timing	Continuous
Weekend midpoint time	47	6.12	Timing	Continuous
Weekend wake time	18	2.34	Timing	Continuous

Statistically significant associations are defined as FDR-corrected *p*-value, derived using the Benjamini-Hochberg method to control false discovery rate (FDR) for multiple testing across all metabolites within each model and sex stratum for each sleep phenotype is below 0.05; while the unadjusted two-sided *p* values were derived by accounting for the complex sampling design-based degrees of freedom, using adjusted standard errors to compute the t-statistic in single metabolite association analysis with each sleep phenotype as dependent variables.

Supplementary Figure S2 visualises the strength of associations (i.e., negative logarithm of the FDR-corrected *p*) between sleep phenotypes and individual metabolites grouped by superpathway. Compared to the sex-stratified analysis, sex-combined analysis tended to identify more statistically significant associations between sleep phenotypes and metabolites (Supplementary Figure S3), in accordance with the higher power due to larger sample size of the sex-combined analysis.

The results from Model 2 were largely consistent with Model 1 results—less than 0.02% (6 out of 30,720) metabolite-sleep phenotype pairs resulted in effect estimates falling outside of the 95% CI of Model 1 effect estimates once controlling for additional lifestyle covariates (Supplemental Table S4). However, only two of these associations were statistically significant in the primary Model 1 analysis and none were significant in the Model 2 analysis. Both associations were with the same unnamed metabolites, and two self-reported insomnia phenotypes.

Sensitivity analysis comparing the aggregation of data across the two batches (as in the primary analysis) to separate analysis in each batch followed by meta-analysis resulted in highly correlated effect sizes across the seven compared sleep-related phenotypes (Supplementary Figure S4), suggesting that it is appropriate to aggregate data across batches while adjusting for batch as a covariate.

Results from analysis of the potential of individual metabolites to serve as biomarkers are reported in Supplementary Table S5. In essence, none of the metabolites explained a large proportion of the variance of the sleep-related phenotype, either individually, or on top of baseline covariates, as assessed using the adjusted R squared. The largest R squared observed using an individual metabolite, without accounting for other covariates, was glutamate, which had adjusted R squared of 0.06 in association with self-reported snoring. The next highest was isovaleryl carnitine (C5), with adjusted R squared of 0.04 in association with REI3%. The largest increase in adjusted R squared was observed for 1-(1-enyl-palmitoyl)-2-oleoyl-GPC (P-16:0/18:1), in association with average HR during sleep. It increased the adjusted R squared from 0.12 in the base model, to 0.14 in the model that included this metabolite.

### Aggregating association analysis results by pathway and by domain

Among the 768 included metabolites, carbohydrates had the highest average number of statistically significant associations with sleep phenotypes (mean: 3.31, range: 1–6 per metabolite), followed by cofactors and vitamins (mean: 3.19, range 0–9 per metabolite) and lipids (mean: 2.53, range: 0–12 per metabolite). Partially characterised molecules (mean: 1.22, range: 0–5) and xenobiotics (mean: 1.4 range: 0–9) had the lowest number of statistically significant associations with sleep phenotypes per metabolite (Supplementary Table S6). The grouping of sleep phenotypes into domains was supported by the correlation matrix based on the effect estimates from the single metabolite association analysis results (Supplementary Figures S5–S7 for sex combined and sex-stratified results) (Fig. 1).

**Fig. 2:**
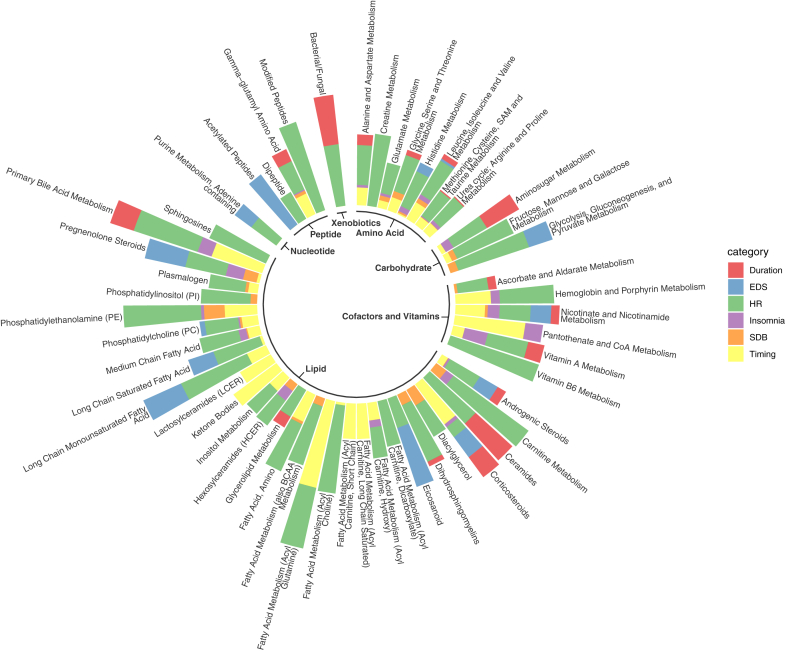
**Number of statistically significant associations between metabolites and sleep phenotypes aggregated by subpathway and sleep phenotype domain**. Statistically significant associations are defined as FDR-corrected *p*-value, derived using the Benjamini-Hochberg method to control false discovery rate (FDR) for multiple testing across all metabolites within each model and sex stratum for each sleep phenotype is below 0.05; while the unadjusted two-sided *p* values were derived by accounting for the complex sampling design-based degrees of freedom, using adjusted standard errors to compute the t-statistic in single metabolite association analysis with each sleep phenotype as dependent variables. The sample sizes for the association analysis of each pair of metabolite and sleep phenotype are available in figshare (doi.org/10.6084/m9.figshare.27212511).

We aggregated metabolites by subpathway and sleep phenotypes by domain, then identified and visualised top metabolomic subpathways with over 25% statistically significant associations (defined as FDR-corrected *p* < 0.05) among all tested associations (Fig. 2) by sleep phenotype domain. Primary bile acid metabolism showed the highest cumulative percentage of statistically significant associations across four sleep phenotype domains, although no significant association was identified for SDB and EDS phenotypes (Supplementary Table S7). Subpathways from amino acid metabolism—specifically leucine, isoleucine, valine, phenylalanine, and tyrosine metabolism—as well as nicotinate and nicotinamide metabolism (cofactors and vitamins), and androgenic steroid metabolism (lipids), showed statistically significant associations with sleep phenotypes across all six domains (Supplementary Table S7). Both lipids and cofactors/vitamins had subpathways (e.g., ketone bodies, acyl glutamine, pantothenate and CoA metabolism, nicotinate and nicotinamide metabolism) that had high percentage of statistically significant associations with sleep timing phenotypes. SDB phenotypes had the highest percentage of statistically significant associations among lipids, especially phosphatidylethanolamine (PE), diacylglycerols, progestin steroids, lysoplasmalogen, and pregnenolone steroids. As for the sleep duration domain, the top subpathways with the highest percentage of significant associations were ceramides, bacterial/fungal, amino sugar metabolism, polyamine metabolism, oxidative phosphorylation, corticosteroids, and primary bile acid metabolism. Self-reported insomnia symptoms domain generally had fewer statistically significant associations, among which vitamin A metabolism showed the highest percentage of statistically significant associations. Eicosanoid, acetylated peptides, long chain monounsaturated fatty acid (LCMUFA) and pregnenolone steroids showed the highest percentage of statistically significant associations with EDS.

Comparing men and women, potential sex differences can be observed (Supplementary Figure S8), with the limitation that the sample size of male participants was lower. Relatively more subpathways had statistically significant associations with SDB phenotypes among females compared to males (these include progestin steroids, oxidative phosphorylation, lactosylceramides (LCER) and sphingomyelins). The subpathways from which metabolites were associated with self-reported insomnia symptoms were mostly progestin steroids, amino sugar metabolism among males, as compared to pregnenolone steroids among females. 13 subpathways, mostly lipids, were significantly associated with EDS among males while no metabolite were identified for EDS in the female-only analysis.

**Fig. 3:**
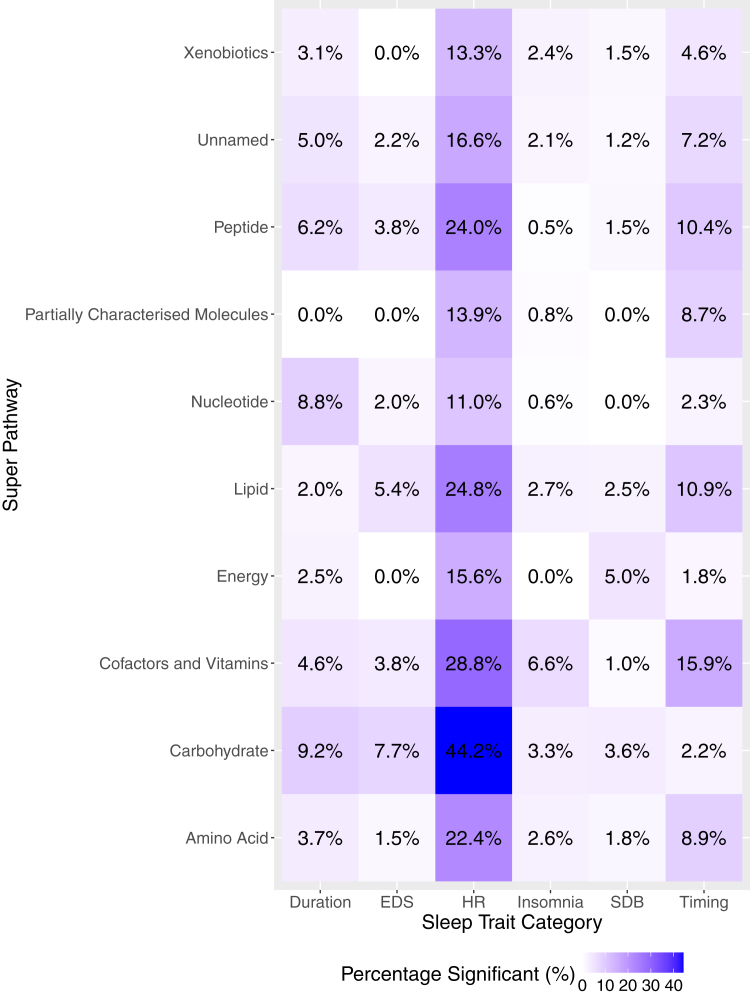
**Percentage of statistically significant associations between metabolites and sleep phenotypes aggregated by superpathway and sleep phenotype domain**. Statistically significant associations are defined as FDR-corrected *p*-value, derived using the Benjamini-Hochberg method to control false discovery rate (FDR) for multiple testing across all metabolites within each model and sex stratum for each sleep phenotype is below 0.05; while the unadjusted two-sided *p* values were derived by accounting for the complex sampling design-based degrees of freedom, using adjusted standard errors to compute the t-statistic in single metabolite association analysis with each sleep phenotype as dependent variables. The sample sizes for the association analysis of each pair of metabolite and sleep phenotype are available in figshare (doi.org/10.6084/m9.figshare.27212511).

We also aggregated metabolites by superpathway and visualised the percentage of statistically significant associations between superpathways and sleep phenotype domains in the format of a heatmap (Fig. 3). The percentage of statistically significant associations were highest for HR during sleep, especially among carbohydrates (44.2%), followed by cofactors and vitamins (28.8%), lipids (24.8%) and peptides (24.0%) (Supplementary Table S8). Self-reported insomnia and SDB domain showed overall lower percentage of statistically significant associations with metabolites across all superpathways, all of which were below 7%.

**Fig. 4:**
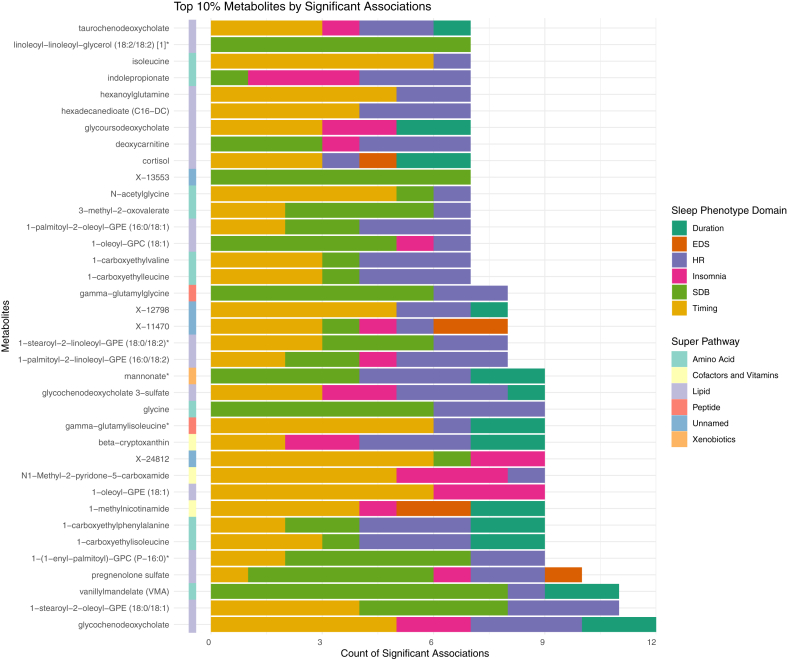
**Number of significant associations aggregated by sleep phenotype domain among the top 10% connected metabolites with the most statistically significant associations**. Statistically significant associations are defined as FDR-corrected *p*-value, derived using the Benjamini-Hochberg method to control false discovery rate (FDR) for multiple testing across all metabolites within each model and sex stratum for each sleep phenotype is below 0.05; while the unadjusted two-sided *p* values were derived by accounting for the complex sampling design-based degrees of freedom, using adjusted standard errors to compute the t-statistic in single metabolite association analysis with each sleep phenotype as dependent variables. The sample sizes for the association analysis of each pair of metabolite and sleep phenotype are available in figshare (doi.org/10.6084/m9.figshare.27212511).

Among all metabolites associated with at least one sleep phenotype, we identified the top 10% (n = 37) with the highest number of total associations, regardless of domains (Fig. 4). 17 metabolites were lipids, 12 were amino acids, 4 were unnamed, 3 were cofactors and vitamins, 2 were peptides, and 1 was xenobiotic. Majority of the top 10% metabolite had associations with more than one sleep phenotype domain (except for linoleoyl-linoleoyl-glycerol (18:2/18:2) [1]∗ and X-13553), among which 10 metabolites were associated with four or more domains of sleep phenotypes. Glycochenodeoxycholate (primary bile acid metabolism), vanillyl mandelate (VMA) and 1-stearoyl-2-oleoyl-GPE (18:0/18:1) were associated with the highest number of sleep phenotypes; while pregnenolone sulfate was associated with all sleep phenotype domains except for sleep duration (Fig. 4).

**Fig. 5:**
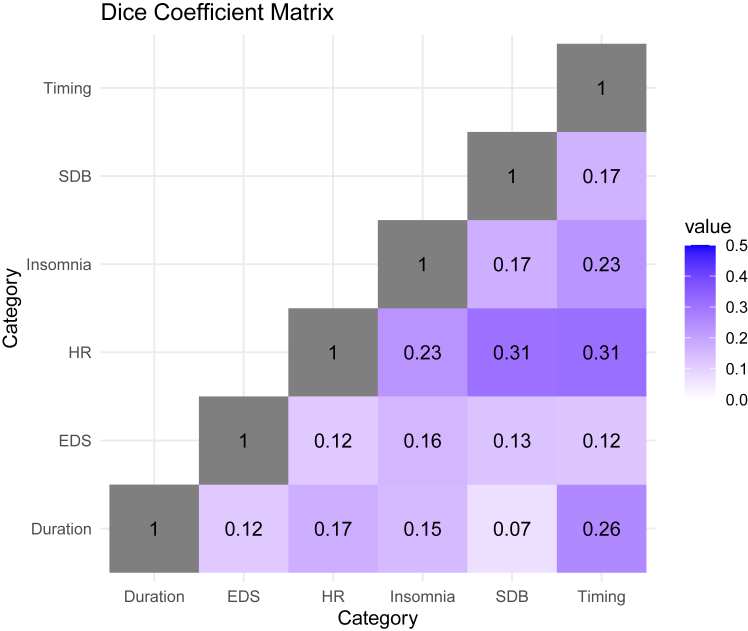
**Dice coefficient matrix of sleep phenotype domain based on associated metabolites**. Dice coefficient was calculated based on the shared associations with metabolites between any two sleep-related phenotype domain over the total significant associations for both sleep phenotype domains combined. A high value (maximum value of 1) indicates complete overlap, while a low value (minimum value of 0) indicates no overlap between two sleep phenotype domains. Statistically significant associations are defined as FDR-corrected *p*-value, derived using the Benjamini-Hochberg method to control false discovery rate (FDR) for multiple testing across all metabolites within each model and sex stratum for each sleep phenotype is below 0.05; while the unadjusted two-sided *p* values were derived by accounting for the complex sampling design-based degrees of freedom, using adjusted standard errors to compute the t-statistic in single metabolite association analysis with each sleep phenotype as dependent variables. The sample sizes for the association analysis of each pair of metabolite and sleep phenotype are available in figshare (doi.org/10.6084/m9.figshare.27212511).

We also compared the similarities among sleep phenotype domains by calculating the Dice Similarity Coefficients (DSC) which quantifies the associated metabolites overlap between any two sleep domains. As shown in Fig. 5, the highest overlap was observed between HR during sleep phenotypes and SDB (DSC = 0.31), and with sleep timing domain (DSC = 0.31), respectively. The overlap between sleep duration and sleep timing domain were lower (DSC = 0.26). The lowest levels of overlap were observed for sleep duration traits and SDB domain (DSC = 0.07).

### Bipartite network analysis based on statistically significant associations between metabolites and sleep phenotypes

**Fig. 6:**
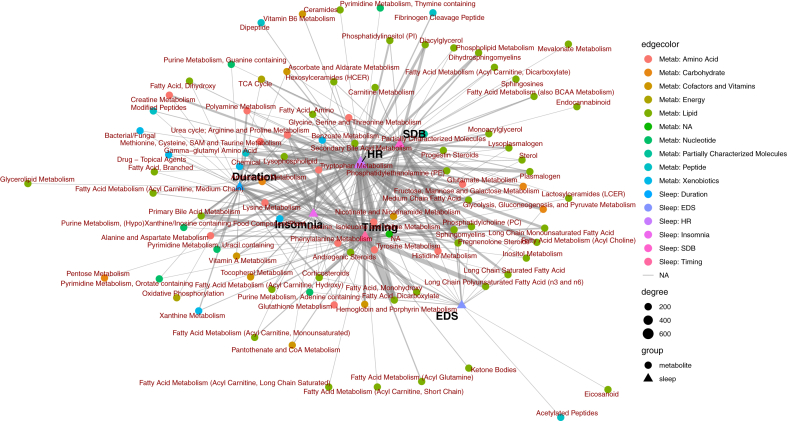
**Network based on associations between metabolites and sleep phenotypes aggregated by subpathway and sleep phenotype domain using the Fruchterman-Reingold force-directed algorithm**. Here we present a network of nodes and edges in which each node represents either a sleep phenotype domain or a metabolomic subpathway, while each edge represents one or more statistically significant associations between two nodes based on the single metabolite association analysis results. The width of the edge (i.e., degree) indicates the number of statistically significant associations between two nodes. The placement of nodes is based on connectivity similarity between nodes, where similar connectivity is reflected as proximity of nodes. Statistically significant associations are defined as FDR-corrected *p*-value, derived using the Benjamini-Hochberg method to control false discovery rate (FDR) for multiple testing across all metabolites within each model and sex stratum for each sleep phenotype is below 0.05; while the unadjusted two-sided *p* values were derived by accounting for the complex sampling design-based degrees of freedom, using adjusted standard errors to compute the t-statistic in single metabolite association analysis with each sleep phenotype as dependent variables. The sample sizes for the association analysis of each pair of metabolite and sleep phenotype are available in figshare (doi.org/10.6084/m9.figshare.27212511).

Fig. 6 shows a network of nodes and edges in which each node represents either a sleep phenotype domain or a metabolomic subpathway, while each edge represents one or more statistically significant associations between two nodes based on the single metabolite association analysis results (FDR-corrected *p* < 0.05). The width of the edge indicates the number of statistically significant associations between two nodes. Some metabolomic subpathways have exclusive connections with one sleep domain, such as fatty acid and dicarboxylate subpathways and sleep timing domain. Others tend to have many connections with multiple sleep domains, such as sphingomyelins with sleep timing, heart rate and SDB phenotype domains. The placement of the nodes reflects their connectivity similarities—the closer two nodes are, the more similar their overall connectivity patterns are. SDB and heart rate phenotypes, for instance, are closely placed together which also corresponds to the high DSC values between the two groups (Fig. 5). Metabolomic subpathways located close to the centre of the network indicates their connectivity with multiple sleep domains, while metabolomic subpathways located at the outskirt of the network indicates their connections with one or few closely located sleep phenotype domains. For instance, metabolites from nicotinate and nicotinamide metabolism and leucine, isoleucine and valine metabolism were associated with all sleep phenotype domains. Lipids (clustered on the right side of the network diagram), were more connected to SDB, HR, sleep timing and EDS than to sleep duration and self-reported insomnia symptoms (Fig. 6), which share similar connectivity patterns with glutamate metabolism, glycolysis, gluconeogenesis, and pyruvate metabolism fructose, mannose and galactose metabolism from amino acid and carbohydrate superpathways.

Treated as a bipartite network model, we further characterised the connectivity between the metabolites, grouped at the superpathway level, and sleep domains quantified by network properties. The number of connected metabolites is the lowest for energy metabolites and the highest for lipids, which corresponds to the number of connected sleep phenotypes for the two superpathways and the average connection per node (i.e., links per node) (Supplementary Figure S9). Similar trend can be observed for the mean number of shared metabolites among sleep phenotypes—the number of overlapping metabolites for sleep phenotypes is the highest among lipids and lowest among energy metabolites, which suggests that more lipids are connected to the same sleep phenotype compared to metabolites from other superpathways. The mean number of shared sleep phenotypes among metabolites, however, peaked for cofactors and vitamins and carbohydrates, followed by lipids, suggesting metabolites from the former two superpathways are more likely to connected to similar sleep phenotypes than other superpathways, despite their low number of total metabolites.

Cluster coefficient, the number of realised links divided by the number of possible links, is the highest among carbohydrates and energy. This pattern indicates a higher degree of overlap in the neighbouring nodes among carbohydrates and energy, compared to unnamed metabolites and when combining all metabolites together regardless of superpathways, which may suggest a lower degree of diversity in terms of their connectivity patterns.

Nestedness of a graph is the property where groups of connected nodes are nested within larger groups of connected nodes. Two versions of nestedness measures were calculated. Both weighted (using the number of sleep domain-superpathway associations) and non-weighted nestedness measures generally agree, and suggest relatively high nestedness among lipids, amino acids, unnamed metabolites as well as combining all metabolites together, and relatively low nestedness among energy, carbohydrates and nucleotides (Supplementary Table S9).

Modularity Q is a measure of community structure. Modulatory values are higher when there are more clusters of connected nodes in a graph. Modularity Q was the highest among xenobiotics (Q = 0.58) and energy (Q = 0.56), followed by peptides (Q = 0.54), and the lowest among carbohydrates (Q = 0.41), suggesting that among the former three superpathways, there are groups of metabolites in the superpathway that tend to connect to the same sleep phenotype. Among carbohydrates, there are fewer such “communities” of metabolites with similar sleep domain connections.

## Discussion

In this study we performed single metabolite association analyses for a variety of sleep phenotypes including SDB, insomnia, EDS, sleep duration, sleep timing, and heart rate measures during sleep, adjusted for common demographic covariates and BMI, and identified metabolites with statistically significant associations with each sleep phenotype. Individually, none of the metabolites were strong biomarker candidates for the studied phenotypes. Taking a network analysis approach, we developed a bipartite network based on the identified associations between metabolites and sleep phenotypes. This approach characterises the connectivity patterns of metabolites from different metabolite super- and sub-pathways, and identifies “neighbouring” metabolites and sleep phenotypes that are inter-connected, as well as metabolomic subpathways that may be of interest for further study.

Many of the metabolites identified in our study as associated with sleep phenotypes have been previously reported to be linked to various sleep phenotypes, usually concurring with our findings. Among the top 10% of connected metabolites with the highest number of associations, regardless of sleep phenotype domain, almost all metabolites were reported in prior publications (Table 3), of which 18 were related to sleep. These include some published results from our prior work in HCHS/SOL (i.e., non-independent findings). Glycine, for instance, has shown associations with multiple SDB phenotypes, and was reported to be associated with sleep deprivation.^44^^,^^75^ In a recent study, glycine was found to be depleted in blood among Hispanic/Latino individuals with severe SDB, likely due to microbiome changes in an oxygen-poor environment.^45^ Four other metabolites – 1-oleoyl-GPE (18:1), 1-(1-enyl-palmitoyl)-GPC (P-16:0)∗, linoleoyl-linoleoyl-glycerol (18:2/18:2) [1]∗ and pregnenolone sulfate, previously reported to be associated with novel SDB phenotype metrics after dimension reduction in our prior work in HCHS/SOL,^63^ were here also associated with several SDB phenotypes as well as with phenotypes from domains including insomnia, sleep timing, and heart rate during sleep.

**Table 3:** Top sleep-connected metabolites and their relevant previously reported associations.

Metabolite	Super pathway	Sub pathway	Prior publications
Glycine	Amino Acid	Glycine, Serine and Threonine Metabolism	Sleep deprivation,^44^ OSA^45^
N-acetyl glycine	Amino Acid	Glycine, Serine and Threonine Metabolism	Sleep restriction^46^
N-lactoyl isoleucine	Amino Acid	Leucine, Isoleucine and Valine Metabolism	Early onset of T2D,^47^ mitochondrial disease^48^
N-lactoyl leucine	Amino Acid	Leucine, Isoleucine and Valine Metabolism	Diabetic retinopathy,^49^ mitochondrial disease^48^
N-lactoyl valine	Amino Acid	Leucine, Isoleucine and Valine Metabolism	Early onset of T2D,^47^ mitochondrial disease^48^
3-methyl-2-oxovalerate	Amino Acid	Leucine, Isoleucine and Valine Metabolism	Impaired fasting glucose^50^
Isoleucine	Amino Acid	Leucine, Isoleucine and Valine Metabolism	Sleep disturbances, circadian rhythms, DM, CVD, obesity^51^
N-lactoyl phenylalanine	Amino Acid	Phenylalanine Metabolism	Metformin, body weight and food intake,^37^^,^^52^ mitochondrial disease^48^
Indole propionate	Amino Acid	Tryptophan Metabolism	T2D^53^^,^^54^
Vanillyl mandelate (VMA)	Amino Acid	Tyrosine Metabolism	Sleep deprivation^55^
1-methylnicotinamide	Cofactors and Vitamins	Nicotinate and Nicotinamide Metabolism	Sleep restriction^56^
N1-Methyl-2-pyridone-5-carboxamide	Cofactors and Vitamins	Nicotinate and Nicotinamide Metabolism	Sleep restriction^56^
Beta-cryptoxanthin	Cofactors and Vitamins	Vitamin A Metabolism	Sleep duration,^57^ sleepiness and sleep disturbance^58^ cognitive function^59^
Deoxycarnitine	Lipid	Carnitine Metabolism	Cognitive impairment and dementia^60^
Cortisol	Lipid	Corticosteroids	Night time awakenings,^61^ CPAP treatment^62^
Linoleoyl-linoleoyl-glycerol (18:2/18:2) [1]∗	Lipid	Diacylglycerol	SDB^63^
Hexanoyl glutamine	Lipid	Fatty Acid Metabolism (Acyl Glutamine)	chronic fatigue syndrome^64^
Hexadecanedioate (C16-DC)	Lipid	Fatty Acid, Dicarboxylate	Blood pressure regulation,^65^ neurodegenerative diseases^66^
1-oleoyl-GPE (18:1)	Lipid	Lysophospholipid	SDB^63^
1-(1-enyl-palmitoyl)-GPC (P-16:0)∗	Lipid	Lysoplasmalogen	SDB^63^
1-palmitoyl-2-linoleoyl-GPE (16:0/18:2)	Lipid	Phosphatidylethanolamine (PE)	Infant sepsis^67^
1-palmitoyl-2-oleoyl-GPE (16:0/18:1)	Lipid	Phosphatidylethanolamine (PE)	Blood pressure^68^
1-stearoyl-2-linoleoyl-GPE (18:0/18:2)∗	Lipid	Phosphatidylethanolamine (PE)	
1-stearoyl-2-oleoyl-GPE (18:0/18:1)	Lipid	Phosphatidylethanolamine (PE)	OSA^24^
Pregnenolone sulfate	Lipid	Pregnenolone Steroids	SDB,^63^ REM sleep^69^
Glycochenodeoxycholate	Lipid	Primary Bile Acid Metabolism	Poor sleep quality^70^
Glycochenodeoxycholate 3-sulfate	Lipid	Primary Bile Acid Metabolism	OSA^29^
Taurochenodeoxycholate	Lipid	Primary Bile Acid Metabolism	OSA,^26^ sleep midpoint^27^
Glycoursodeoxycholate	Lipid	Secondary Bile Acid Metabolism	Bed time^71^
Gamma-glutamyl isoleucine∗	Peptide	Gamma-glutamyl Amino Acid	Sleep midpoint,^27^ wake time and sleep midpoint^72^
Gamma-glutamyl glycine	Peptide	Gamma-glutamyl Amino Acid	Myocardial infarction^73^
Mannonate∗	Xenobiotics	Food Component/Plant	Undernutrition^74^

N-lactoyl isoleucine, N-lactoyl leucine, N-lactoyl valine, N-lactoyl phenylalanine were relabelled from the original annotation (previously 1-carboxyethylisoleucine, 1-carboxyethylleucine, 1-carboxyethylvaline, 1-carboxyethylphenylalanine) based on.^37^

Abbreviations. CPAP: continuous positive airway pressure; OSA: obstructive sleep apnoea; REM: repetitive eye movement; SDB: sleep disordered breathing; T2D: type 2 diabetes.

Many of the metabolites identified in this study were also reported to be associated with comorbidities such as cardiovascular, metabolic, and neurodegenerative diseases, potentially connecting sleep to a wide range of chronic adverse health outcomes. Four metabolites listed in Table 3 belong to the group of N-lactoyl amino acids—specifically, N-lactoyl isoleucine, N-lactoyl phenylalanine, N-lactoyl leucine, and N-lactoyl valine—which were previously misidentified as 1-carboxyethyl amino acids by Metabolon.^37^ N-lactoyl amino acids (lac-AAs) are an emerging class of bioactive metabolites that link amino acid and energy metabolism. They are formed through cytosolic nonspecific dipeptidase 2 (CNDP2)-mediated reverse proteolysis of lactate with amino acids.^76^ Recent studies have associated lac-AAs with various physiological and pathological conditions, including exercise,^77^ prediabetes and diabetes,^47^ appetite suppression,^37^ and poor outcomes in septic shock.^78^ Lac-AAs may link sleep-related phenotypes and metabolic disorders such as obesity and diabetes. Cortisol, as the end product of the hypothalamic-pituitary-adrenal (HPA) axis, has shown to be associated with sleep disturbance in a bidirectional manner,^79^ and linked to increased adiposity, insulin resistance, and dyslipidaemia.^80^ Hexadecanedioate (C16-DC), a dicarboxylate fatty acid, was reported to be associated with blood pressure regulation^65^ and neurodegenerative diseases.^66^ Beta-cryptoxanthin, an antioxidant and pre-vitamin A carotenoid found in fruits and vegetables, was found positively associated with cognition (analysis was not adjusted for sleep traits) in individuals of diverse race/ethnic backgrounds.^59^ These metabolites suggest some shared biochemical mechanisms between sleep and other chronic adverse health outcomes.

When working with complex data such as untargeted metabolomic profiling and multiple phenotypes, effectively summarise data and develop useful insights is challenging.^81^ Different visualisation and analytical approaches have been developed to facilitate this process.^82^ Here, we applied a systems biological approach – network analysis which has been widely used in gene expression, gene regulation, gene-disease network, and drug–drug interaction studies, on statistical relations among multiple phenotypes and metabolites. This data-driven network approach is different from knowledge-based network construction approaches^83^ built on biochemical relations such as KEGG networks.^84^ Here, the network was built on inferred statistical relations among metabolites and phenotypes, leveraging, for interpretation purposes, well-studied network properties metrics from other fields such as ecology, socioeconomics, neuroscience, drug-disease networks, among others.^85^, ^86^, ^87^, ^88^ One interesting observation is the relatively high nestedness among lipids and amino acids. High nestedness indicates that metabolites from these two superpathways form such a structure that metabolites with fewer connections (referred to as “degree”) are more likely to “connect”, via their mutual connections with sleep domains, with metabolites with higher degree of connections, rather than with other metabolites with a similar low degree of connectiveness. An intuitive depiction of such network is a “core-periphery” structure, in which a “core” of nodes is connected with other nodes, where a “periphery” of nodes tends to only connect with the nodes in the core. Contextually, among lipids and amino acids associated with sleep phenotypes, higher nestedness, compared to other superpathways, implies that these superpathways are more likely to have a subset of metabolites that play a “key” role forming connections with many, and the same, sleep phenotypes, rather than an evenly distributed network where metabolites form connections with various sleep phenotypes from different domains in a random manner. Metabolites with high connectiveness from these two super-pathways likely have roles in shared biological processes across multiple sleep phenotypes, especially considering 26 out of 32 top 10% connected metabolites are either amino acids or lipids. Modularity metrics, on the other hand, measure how well a network can be partitioned into clusters or compartments in which there are dense connections internally and sparser connections with other clusters.^89^ Among xenobiotics, energy and peptide metabolites associated with sleep phenotypes, relatively higher modularity compared to other superpathways suggests the existence of such subgroups with distinct relationships with sleep phenotypes.

HR domain had the highest percentage of metabolite associations of these assessed. HR during sleep reflects activity of the autonomic nervous system, and is influenced by cardiac function, sleep stage (i.e., lowest in deep restorative sleep and highest and most variable in REM sleep and wakefulness during the sleep period), and sleep apnea-related heart rate response.^90^^,^^91^ Notably, both low and elevated heart rate response to SDB events are associated with biomarkers of cardiovascular diseases, while elevated heart rate response to SDB events is predictive of incident fatal and non-fatal CVD.^92^ The finding that HR during sleep associated with hundreds of metabolites, from all superpathways, may reflect the multiple biological mechanisms that underlie this phenotype, as well as support the sleep-related HR as a marker of multiple biologically processes that may be targeted for interventions. Sleep timing also was associated with a relatively high number of metabolite associations, supportive of growing data implicating timing-related behaviours on metabolic outcomes,^93^ as well as the correlation of sleep timing with other sleep domains.^94^ In contrast, fewer associations were observed between SDB phenotypes and metabolites. This may be because that SDB measured by traditional metrics such as the Apnea-Hypopnea Index (AHI) may poorly characterise disease that is influenced by multiple mechanistic pathways.^95^

An analytic choice that we made that is worth discussing is missing metabolite data imputation. Here, we imputed missing values of metabolites with no more than 25% missing values. For non-xenobiotic metabolites, we selected the imputation method based on an empirical investigation of the proportion of replicated associations between batches, and the selected method was multiple imputation that included all metabolite and other measures (covariates and lab values) that have strong associations with metabolite levels. It is natural to question whether this may somehow bias results: for example, is it possible that, for instance, using diabetes and BMI (among the rest) to impute metabolite values will lead to metabolite values that are “too reflective” of BMI and diabetes and therefore will somehow generate spurious metabolite associations with sleep measures that are associated with BMI and diabetes? The answer is that this is very unlikely. Generation of metabolite values that are overly similar to BMI or diabetes (in this example) by the predictive mean matching function suggests overfitting to the values of these covariates. Such overfitting will reduce the likelihood of replication of associations in the second batch, rather than increase it. Further, the predictive mean matching has some randomness due to sampling, further limiting overfitting to the variables used in imputation. Finally, it is important to note that the correlation between metabolites and covariates, as with sleep, is an inherent characteristic of this biological signal (i.e. it is a “feature, not a bug”), as are the metabolite associations with sleep measures. Therefore, leveraging this characteristic is useful.

There are several strengths to this study: we looked at multiple sleep phenotypes simultaneously which provides a unique opportunity for pattern recognition across phenotypes. We also explored the network analysis approach in summarising the metabolite-sleep phenotypes associations which enabled us to leverage well developed network property metrics from other fields to offer new insights and potentially lead to more hypothesis generation. Additionally, we compared the metabolite-sleep phenotype associations in combined sexes and sex-stratified study populations, recognising more work is needed to further understand the implications of potential differences. Lastly, the atlas created in this study will be a useful resource for the scientific community. This study also has a few limitations. First, our study population is based on the HCHS/SOL cohort, representative of the Hispanic/Latino population in the US. Although it's important to study under-represented populations such as Hispanic/Latino individuals, further studies on other populations are needed to increase the generalisability of the findings. Second, the network analysis conducted in this study is not to make network inference but to summarise the associations results. Third, due to the large breadth of analyses we did not account for medication use in this work. This can influence associations as medications can affect metabolite levels and sleep phenotypes. Similarly, fourth, our primary model for which we summarise the results was not adjusted for comorbidities. In all, one cannot infer mechanisms and directionality of associations from these analyses. Fifth, another limitation is that the objective overnight sleep measures did not use EEG, limiting SDB (e.g. we do not have measures of arousals) as well as potential sleep staging measures. Sixth, any comparison between the sleep domains used in this analysis is limited by the available sleep phenotypes, their number, and the correlation patterns between them. Finally, the recorded sex data was not verified, and it is possible that the sex of a few individuals was mislabelled.

In summary, we studied the associations between multiple sleep phenotypes from multiple sleep domains and the metabolomic environment in a large population-based cohort study. Using network analysis, we were able to visualise the interconnectedness between multiple sleep phenotypes and associated metabolites simultaneously, which provides an opportunity to glean into connectivity patterns that otherwise might be obscure when presented as individual relationships. We also created a resource for the sleep research community that will facilitate hypothesis generation in future metabolomic studies on sleep health. As sleep is highly affected by the social and built environment, it would be important, in the future, to use metabolomics to glean into the pathways by which the environment impacts sleep.

## Contributors

Ying Z performed association analyses, network analyses, and data visualisation. BWS developed the R/shiny app for visualisation of results from single metabolite association analysis. Yu Z performed data imputation and sensitivity analyses. DAW provided code for modelling circular sleep phenotypes. BY, QQ, and EB designed and established the metabolomics ancillary studies. MA, LA-S, MD, RK, and JC participated in HCHS/SOL study design, recruitment, and operations. Ying Z and TS drafted the manuscript. BWS, Yu Z, DAW, BY, QQ, MA, LA-S, EB, MD, RK, JC, and SR critically reviewed and approved the manuscript. TS supervised the work. Data was accessed and verified by Ying Z, Yu Z, and TS. All authors read and approved the final version of the manuscript.

## Data sharing statement

HCHS/SOL data are available through application to the data base of genotypes and phenotypes (dbGaP) accession phs000810. HCHS/SOL metabolomics data are available via data use agreement with the HCHS/SOL Data Coordinating Center (DCC) at the University of North Carolina at Chapel Hill, see collaborators website: https://sites.cscc.unc.edu/hchs/. The metabolite association data generated in this study are provided in figshare (doi.org/10.6084/m9.figshare.27212511) and available for interactive data visualisation via a shiny app https://bidmc-cardiology-2024.shinyapps.io/sleep_metabolite_viewer_v1/. The code used in this work has been deposited in the public repository https://github.com/yzhang104/HCHS_SOL_Sleep_Metabolomics_Atlas.git.

## Declaration of interests

Dr. Argos reports receiving grants R01ES033883, U01HG013275, U2RTW010122, and R01CA255082, with payments made to the institution. Dr. Redline reports receiving consultant fees from Eli Lilly, with payments made to self, unpaid participation in the Data Safety Monitoring Board or Advisory Board of Apnimed, and unpaid leadership or fiduciary roles in the Alliance for Sleep Apnea Partners and in the National Sleep Foundation. Dr. Sofer reports receiving a grant from the National Human Genome Research Institute, with payments made to the institution.

## Appendix A Supplementary data

Supplementary Notes and Figures
