# Supplementary material for: Untargeted metabolome atlas for sleep-related phenotypes in the Hispanic community health study/study of Latinos
Source: eBioMedicine. 2024 Dec 17;111:105507. doi: 10.1016/j.ebiom.2024.105507 (PMC11722176; doi:10.1016/j.ebiom.2024.105507)
Supplement: Supplementary Notes and Figures [file mmc1.docx]

Untargeted Metabolome Atlas for Sleep Phenotypes in the Hispanic Community Health Study/Study of Latinos:
Supplementary Information

[Supplementary Note 1: Sleep phenotypes used. 1](#_Toc179365974)

[Supplementary Note 2: Assessment of metabolite imputation strategy. 3](#_Toc179365975)

[Supplementary Figure S1: Study sample selection and pre-processing for the metabolomic analysis 6](#_Toc179365976)

[Supplementary Figure S2. Scatter plot of single metabolite association analysis with sleep phenotypes 7](#_Toc179365977)

[Supplementary Figure S3. Number of statistically significant associations between metabolites and sleep phenotypes by domain and sex strata 9](#_Toc179365978)

[Supplementary Figure S4. Correlation between association effect estimates from batch-aggregated and batch-separate analysis 10](#_Toc179365979)

[Supplementary Figure S5. Correlations between estimated metabolite associations across sleep-related phenotypes (sex-combined). 11](#_Toc179365980)

[Supplementary Figure S6. Correlations between estimated metabolite associations across sleep-related phenotypes (female group). 12](#_Toc179365981)

[Supplementary Figure S7. Correlations between estimated metabolite associations across sleep-related phenotypes (male group). 13](#_Toc179365982)

[Supplementary Figure S8: Number of statistically significant associations between metabolites and sleep phenotypes aggregated by subpathway and sleep phenotype domain stratified by sex 14](#_Toc179365983)

[Supplementary Figure S9. Network structural property metrics summarised by superpathway 15](#_Toc179365984)

[References 16](#_Toc179365985)

# Supplementary Note 1: Sleep phenotypes used.

We used sleep measures assigned into 5 domains: sleep duration, heart rate during sleep, insomnia, sleep disordered breathing, and sleep timing. We provide details about these phenotypes by domain.

**Sleep duration:** questionnaire items included bed time and wake time over the weekdays and weekend days. Based on these, we computed sleep duration during weekdays, sleep duration during weekdays, sleep duration (weighted average of weekdays and weekend sleep duration, with weights being 5/7 and 2/7 for weekdays and weekend days, respectively), and binary variables for short sleep ($\leq5$ hours) during weekdays, and long sleep ($\geq9$ hours) during weekdays.

**Heart rate (HR):** minimum, maximum, average, and standard deviation of heart rate during sleep (all continuous measures).

**Insomnia:** this category included variables measured via the women health initiative insomnia rating scale (WHIIRS), which combines 4 likert scale questions, use of sleeping pills, restless sleep, and excessive daytime sleepiness. Specifically, we included the WHIIRS score as a continuous measure, the Epworth sleepiness scale (ESS; as a continuous measure) and questions dichotomised to binary: typical night's sleep in past 4 weeks (1 if restless or very restless, 0 otherwise), trouble getting back to sleep (1 if 3 or more times a week), wake up earlier than you plan (1 if 3 or more times a week), wake up several times at night (1 if 3 or more times a week), trouble falling asleep (1 if 3 or more times a week), taking sleeping pills (1 if 3 or more times a week), and also excessive daytime sleepiness (EDS) which dichotomised ESS as ESS>10 defining EDS.

**Sleep disordered breathing (SDB):** Multiple measures were based on counts of respiratory events during sleep. Certified polysomnologists manually edited artifacts, identified periods of sleep, and annotated each respiratory event with its associated oxyhaemoglobin desaturation. Respiratory events were identified as a 50% or greater reduction in airflow lasting greater than or equal to 10 seconds. The respiratory event index (REI) is the number of respiratory events per estimated sleep hour, with the REI0 (“all desat”) and REI3 (“3% desat”), comprised of hypopneas scored without a desaturation requirement (REI0) or with associated desaturations of greater than or equal to 3% (REI3). We also used measures based on oxyhaemoglobin saturation (SpO2) during sleep, including related to respiratory events. Specifically:

Continuous measures included REI0, REI3, total count of respiratory events, total length of respiratory events (i.e. the total sleep time with an event), average respiratory event length, hypoxic burden, minimum and average SpO2, percent sleep time with SpO2<90% (perlt90), and dichotomised variables: REI0 and REI3 $\geq5$ and $\geq15$ (each applied with both thresholds), and also self-reported snoring (1 if 6-7 times a week, 0 otherwise).

**Sleep timing:** all these variables were treated as circular, and included weekday wake and , weekend wake and bed time, weekday sleep midpoint, weekend sleep midpoint, and social jetlag, defined as the difference between the sleep midpoint on weekends and on weekdays.

# Supplementary Note 2: Assessment of metabolite imputation strategy.

We compared a few approaches for metabolite imputation and transformation. To decide which approach to use in the analysis, we applied the various approaches on batch 1 and batch 2 metabolite datasets separately, performed association analyses using batch 1, and attempted replication testing of selected metabolites in batch 2. We recorded the number of single metabolite association being replicated in the association models adjusted for age, gender, centre, Hispanic background and BMI. From batch 1 discovery analysis we took metabolites with False Discovery Rate (FDR)-corrected *p* value<0.05 for replication analysis in batch 2. For this investigation, an association was considered replicated in batch 2 if it had p-value<0.05 (unadjusted) in batch 2 analysis. We used the following sleep traits for this analysis: sleep duration, SD of heart rate during sleep, REI3%, midpoint of weekday sleep, wake time of weekday and weekend sleep.

We compared imputation approaches only for non-xenobiotics. For xenobiotics, we applied imputation with half of the minimum values of the corresponding metabolite. Thus, we focused on non-xenobiotic for this analysis. We compared imputation approaches. For all metabolites, and post-imputation transformation (rank-normalisation or no transformation). Compared imputation included:

1. Imputation using half the minimum value observed for the metabolite in the batch.
2. Multiple imputations of the metabolite dataset using a multivariate model with a fully conditional specification (“metaonly”).
3. Multiple imputations of the metabolite and covariates dataset using a multivariate model with a fully conditional specification (“metacov”).
4. Multiple imputations of the metabolite dataset using a multivariate model where each metabolite is imputed using only the top 10 most highly correlated metabolites (“metaonly_UnMet”).
5. Multiple imputations of the metabolite and covariates dataset using a multivariate model where each metabolite is imputed using only the top 10 most highly correlated metabolites and/or covariates (“metacov_UnMet”).

The code used for the “UnMet” methods was taken from [**https://github.com/tofaquih/imputation_of_untargeted_metabolites**](https://github.com/tofaquih/imputation_of_untargeted_metabolites), based on methodology reported in Faquih et al. [^1^](https://sciwheel.com/work/citation?ids=15082067&pre=&suf=&sa=0&dbf=0). All implementations of multiple imputations used 5 imputed datasets, where association analyses were performed in each imputed dataset and later combined. Results are provided in Supplementary Table S10). Multiple imputation using all metabolite values and covariates and fully conditional specification, followed by rank normalisation, was picked as the optimal method and was used in the main analysis which combined the two batches after imputation and rank-normalisation.

# Supplementary Figure S1: Study sample selection and pre-processing for the metabolomic analysis


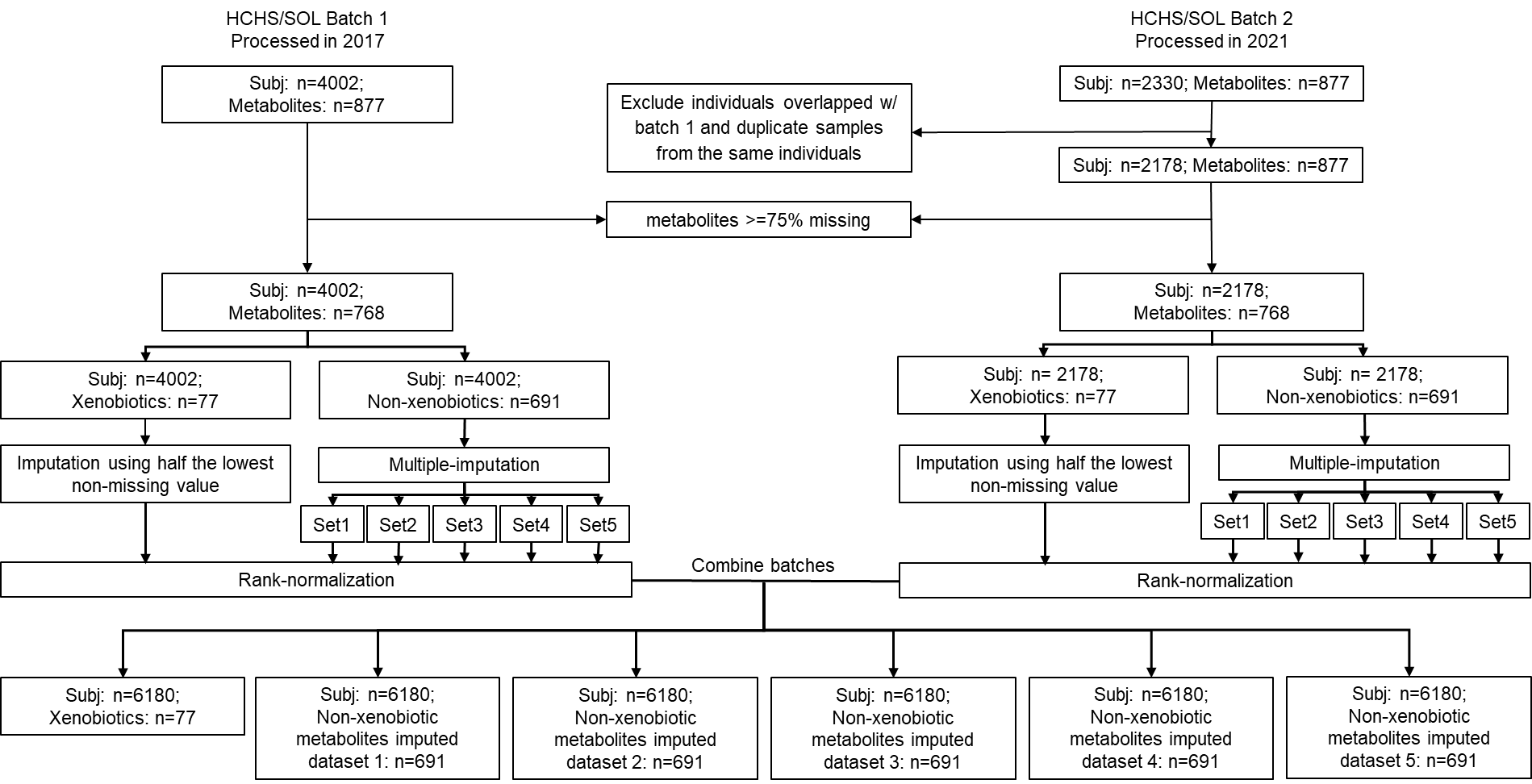


# Supplementary Figure S2. Scatter plot of single metabolite association analysis with sleep phenotypes


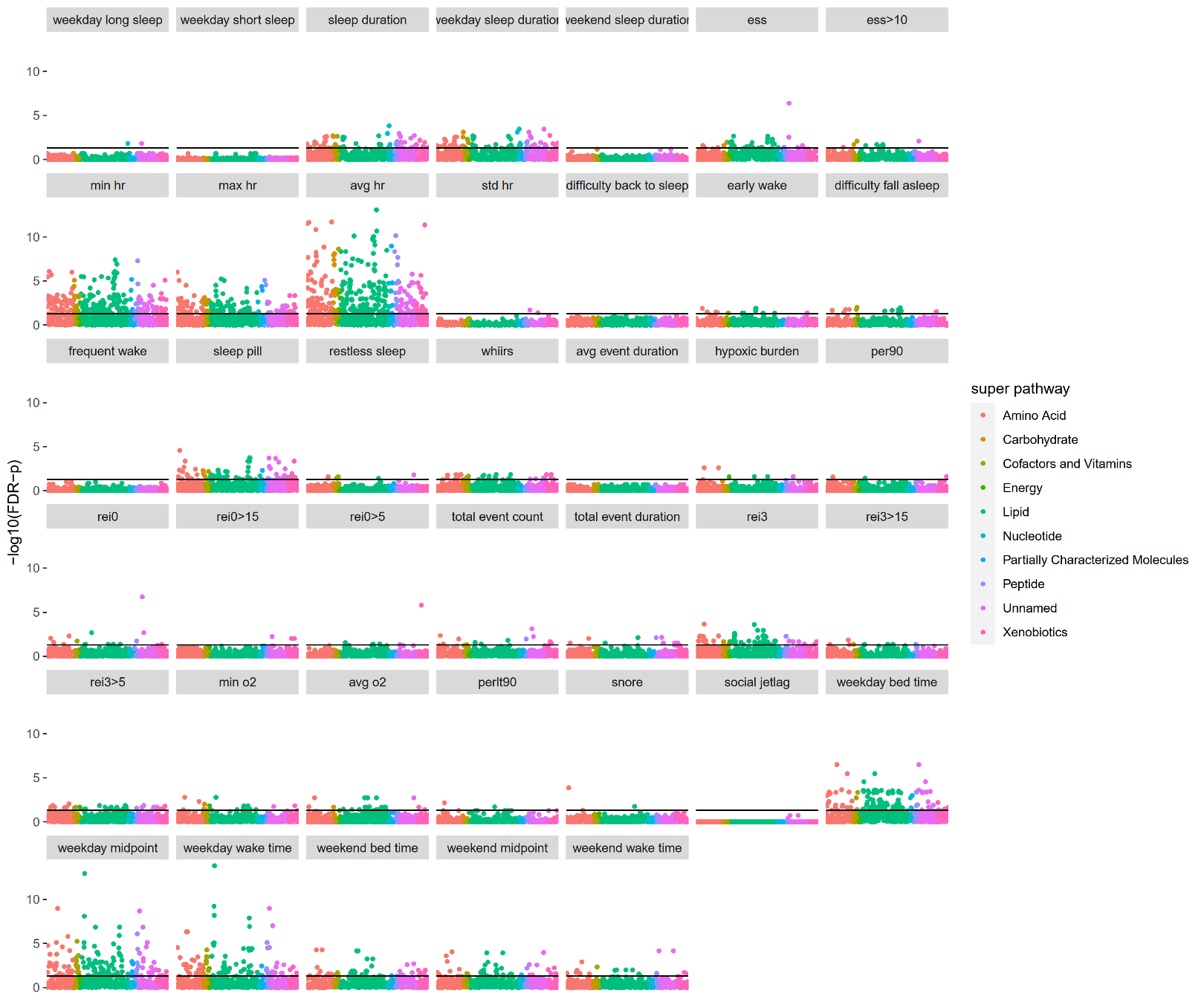

-log10(FDR-*p*) is based on the false discovery rate (FDR)-corrected *p* in which the raw *p* was derived by accounting for the complex sampling design-based degrees of freedom, using adjusted standard errors to compute the t-statistic in single metabolite association analysis with each sleep phenotype as dependent variables, while the FDR adjustment was based on the Benjamini-Hochberg method to control false discovery rate for multiple testing among all metabolites in all models for each sleep phenotype. Black horizontal line indicates FDR-corrected *p*=0.05.

# Supplementary Figure S3. Number of statistically significant associations between metabolites and sleep phenotypes by domain and sex strata


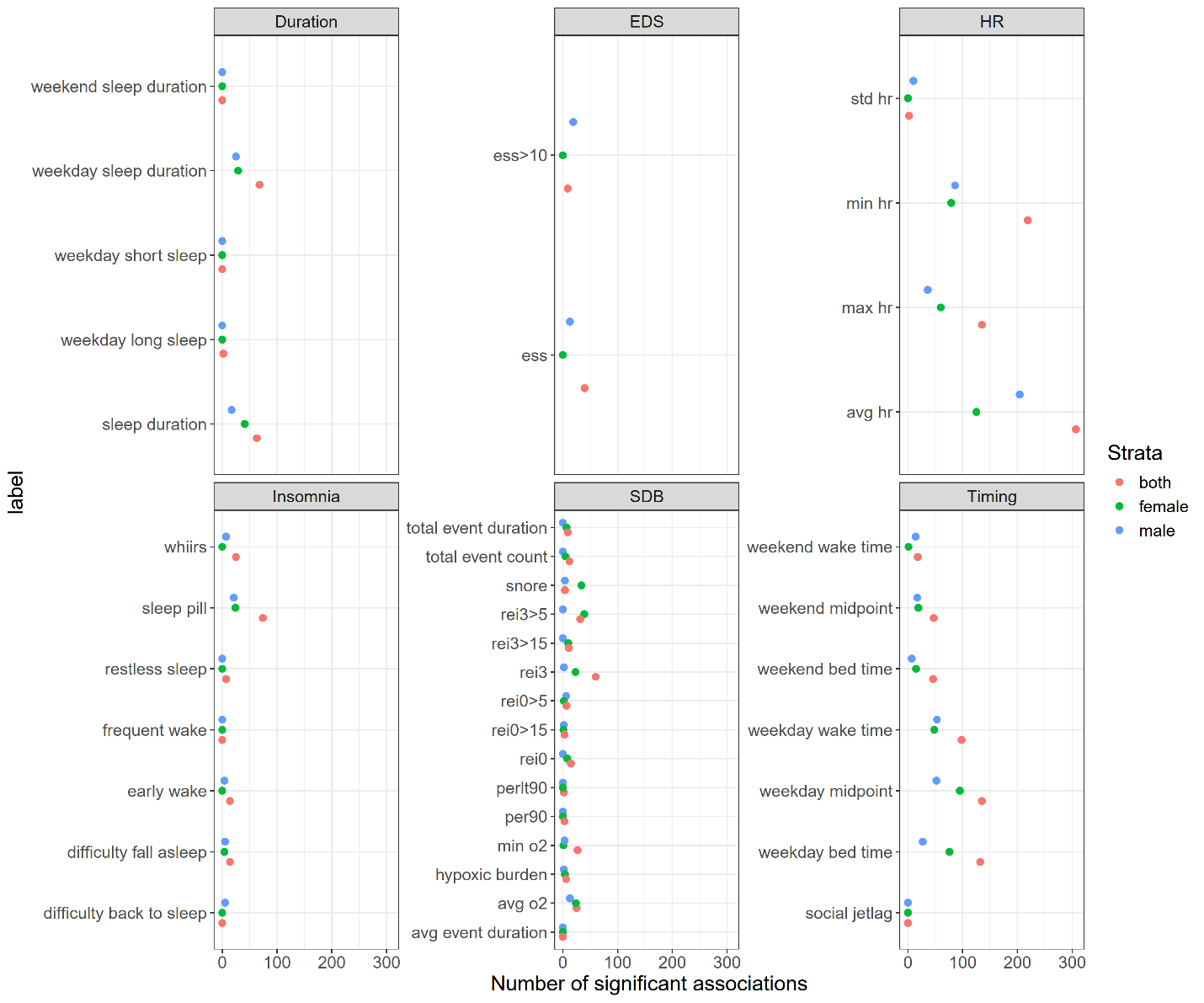


Associations between metabolite and sleep phenotype are identified as statistically significant based on FDR-corrected *p* <0.05. The raw *p*-value was derived by accounting for the complex sampling design-based degrees of freedom, using adjusted standard errors to compute the t-statistic in single metabolite association analysis with each sleep phenotype as dependent variables. The FDR adjustment was based on the Benjamini-Hochberg method to control false discovery rate for multiple testing among all metabolites in all models for each sleep phenotype.

# Supplementary Figure S4. Correlation between association effect estimates from batch-aggregated and batch-separate analysis


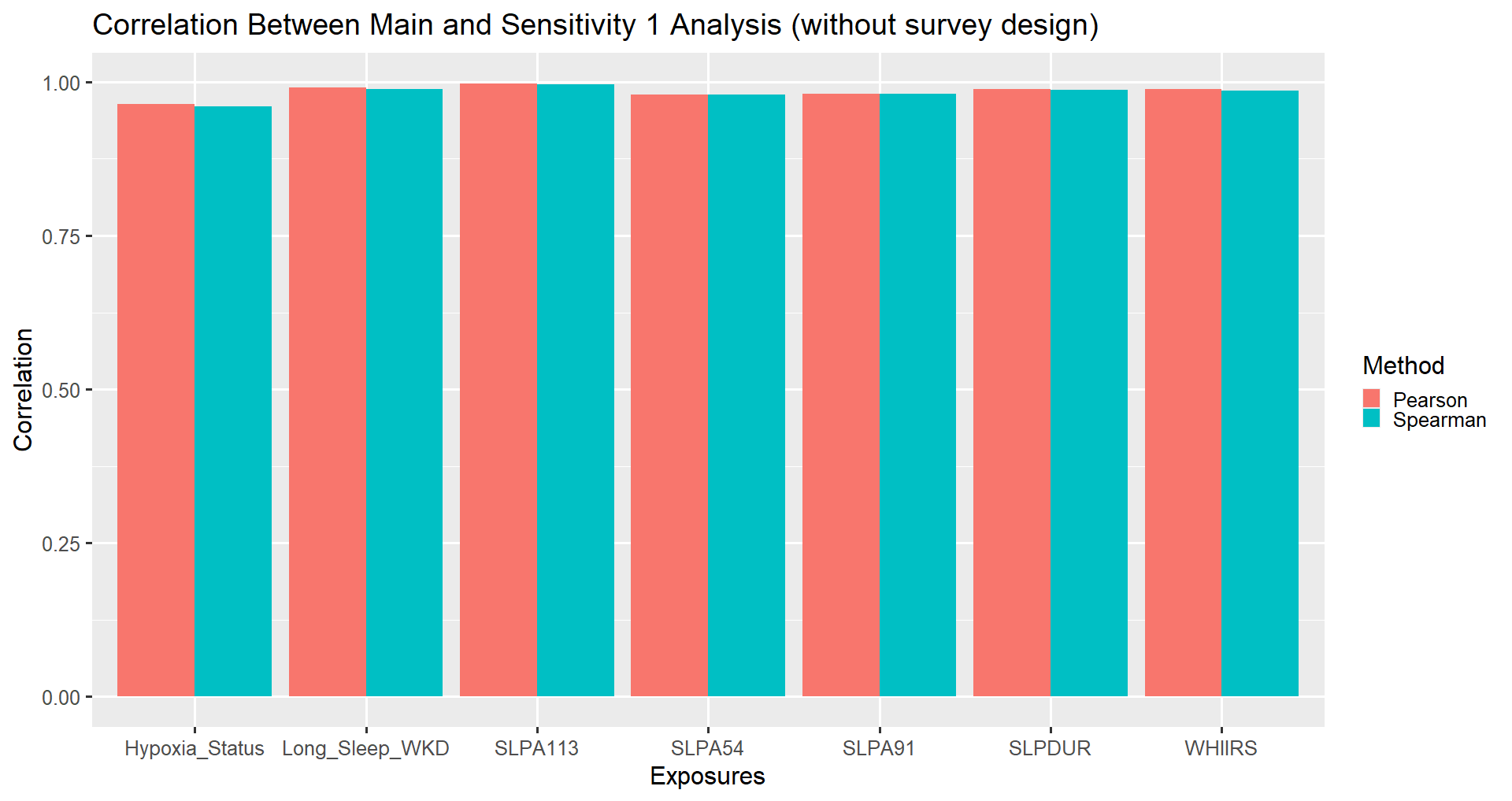


Pearson and Spearman correlations between estimated metabolite associations effect sizes of each of seven phenotypes and all studied metabolites. For each metabolite, both analyses used only measured metabolites (no imputation of missing values). Both analyses used Model 1 covariates (age, sex, BMI, study centre, Hispanic background, and batch indicator for aggregated-batch analysis only). Batch-separate analysis removed 523 individuals from batch 2 who live in the same household as individuals in batch 1. Batch-separate analysis combined results from the two batches in fixed-effects inverse-variance meta-analysis.

# Supplementary Figure S5. Correlations between estimated metabolite associations across sleep-related phenotypes (sex-combined).


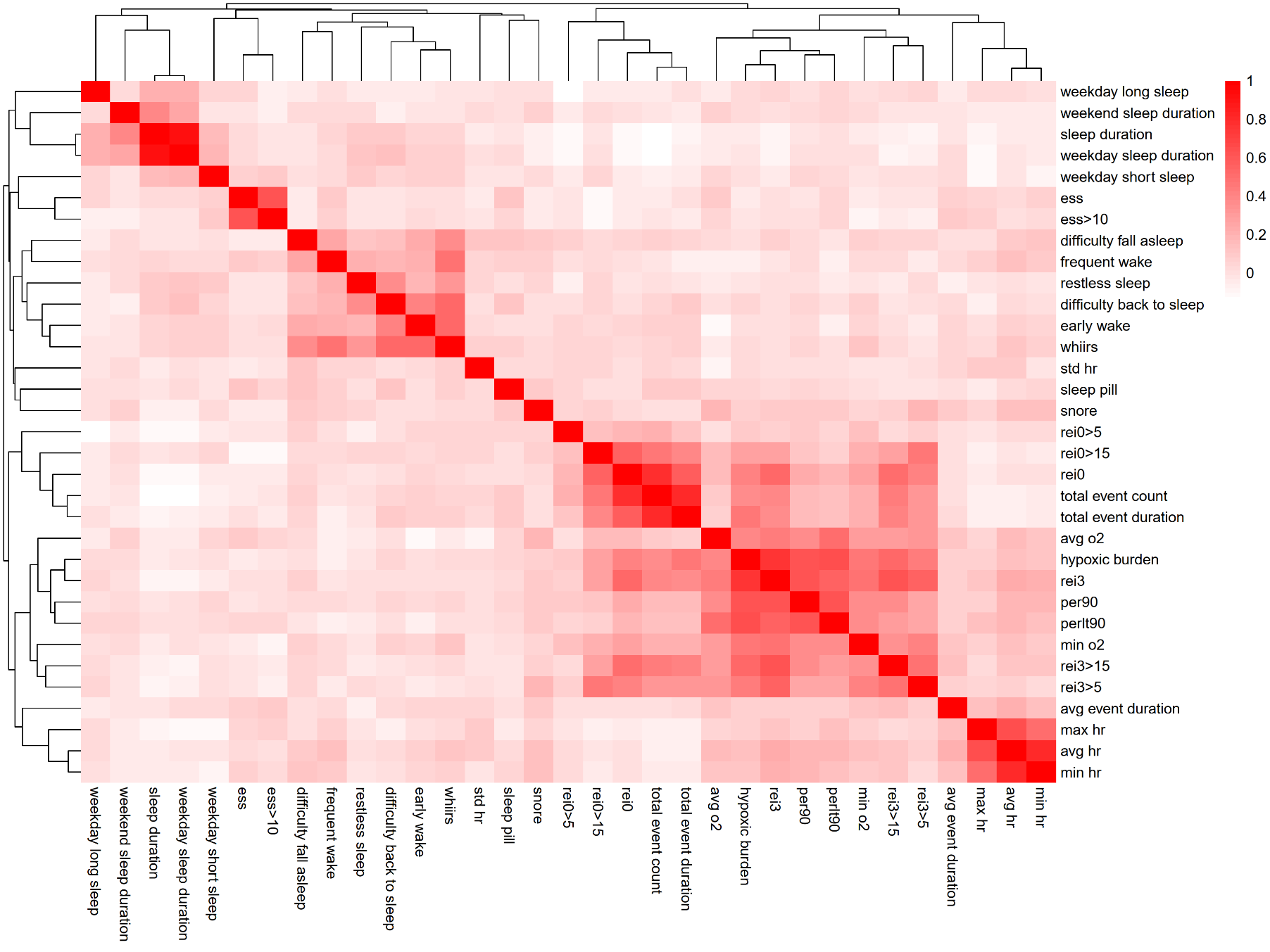


The figure visualises the squared Pearson correlation coefficients across all non-circular sleep- related phenotypes, using Model 1-estimated association effect sizes from all metabolites.

# Supplementary Figure S6. Correlations between estimated metabolite associations across sleep-related phenotypes among females.
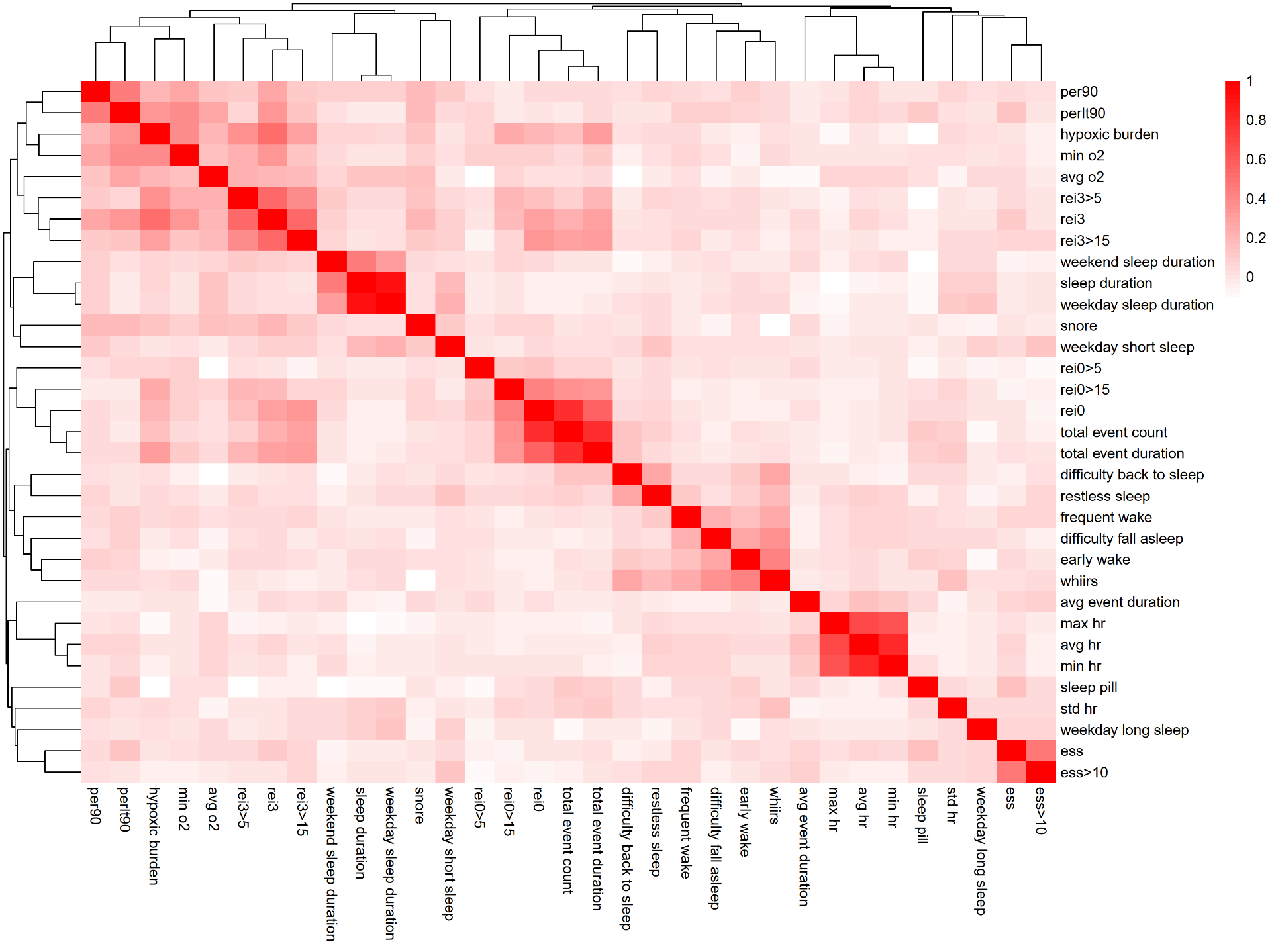


The figure visualises the squared Pearson correlation coefficients across all non-circular sleep- related phenotypes, using Model 1-estimated association effect sizes from all metabolites. Effect size estimates were extracted from female-specific analysis.

# Supplementary Figure S7. Correlations between estimated metabolite associations across sleep-related phenotypes among males
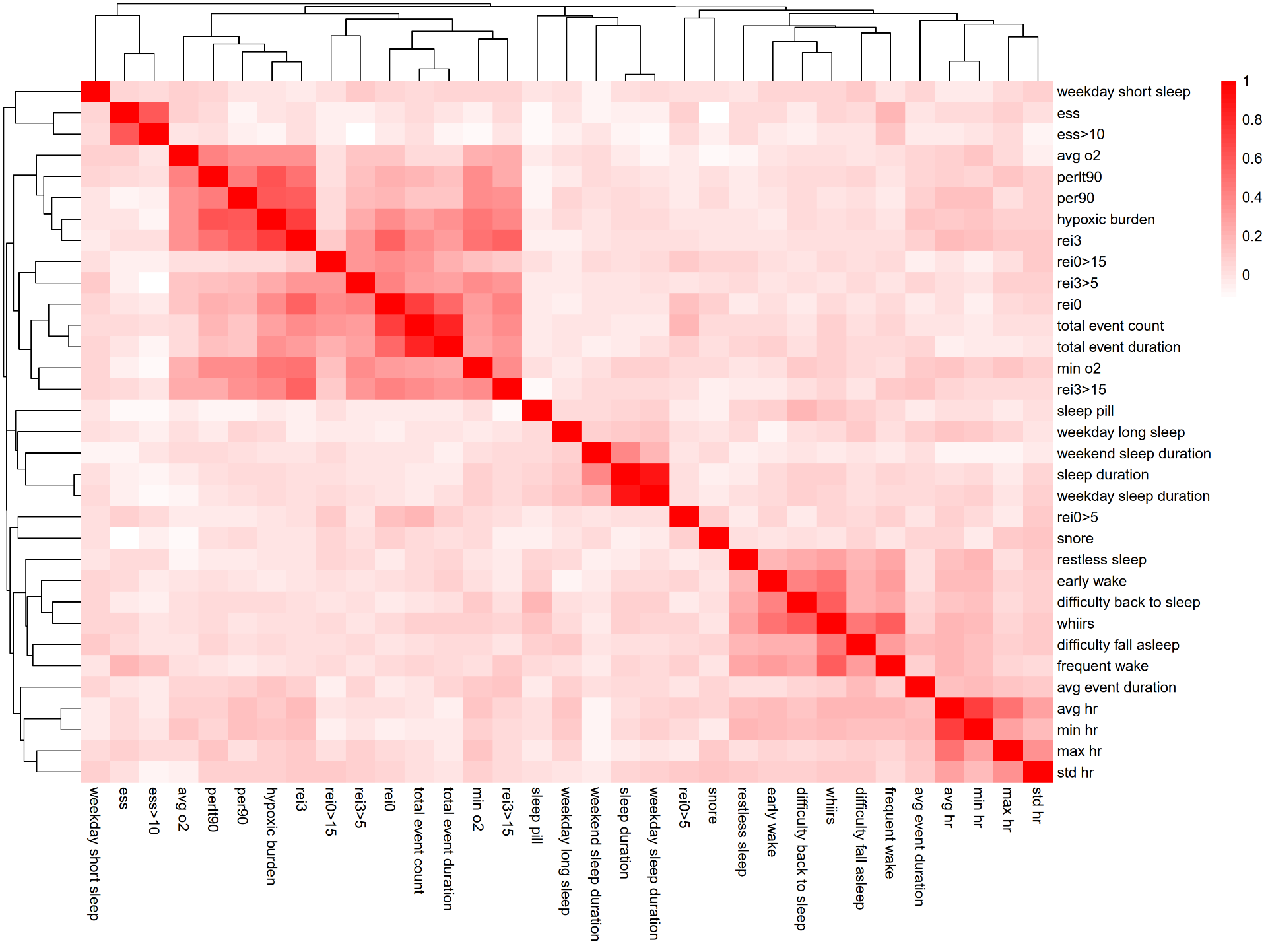


The figure visualises the squared Pearson correlation coefficients across all non-circular sleep- related phenotypes, using Model 1-estimated association effect sizes from all metabolites. Effect size estimates were extracted from male-specific analysis.

# Supplementary Figure S8: Number of statistically significant associations between metabolites and sleep phenotypes aggregated by subpathway and sleep phenotype domain stratified by sex


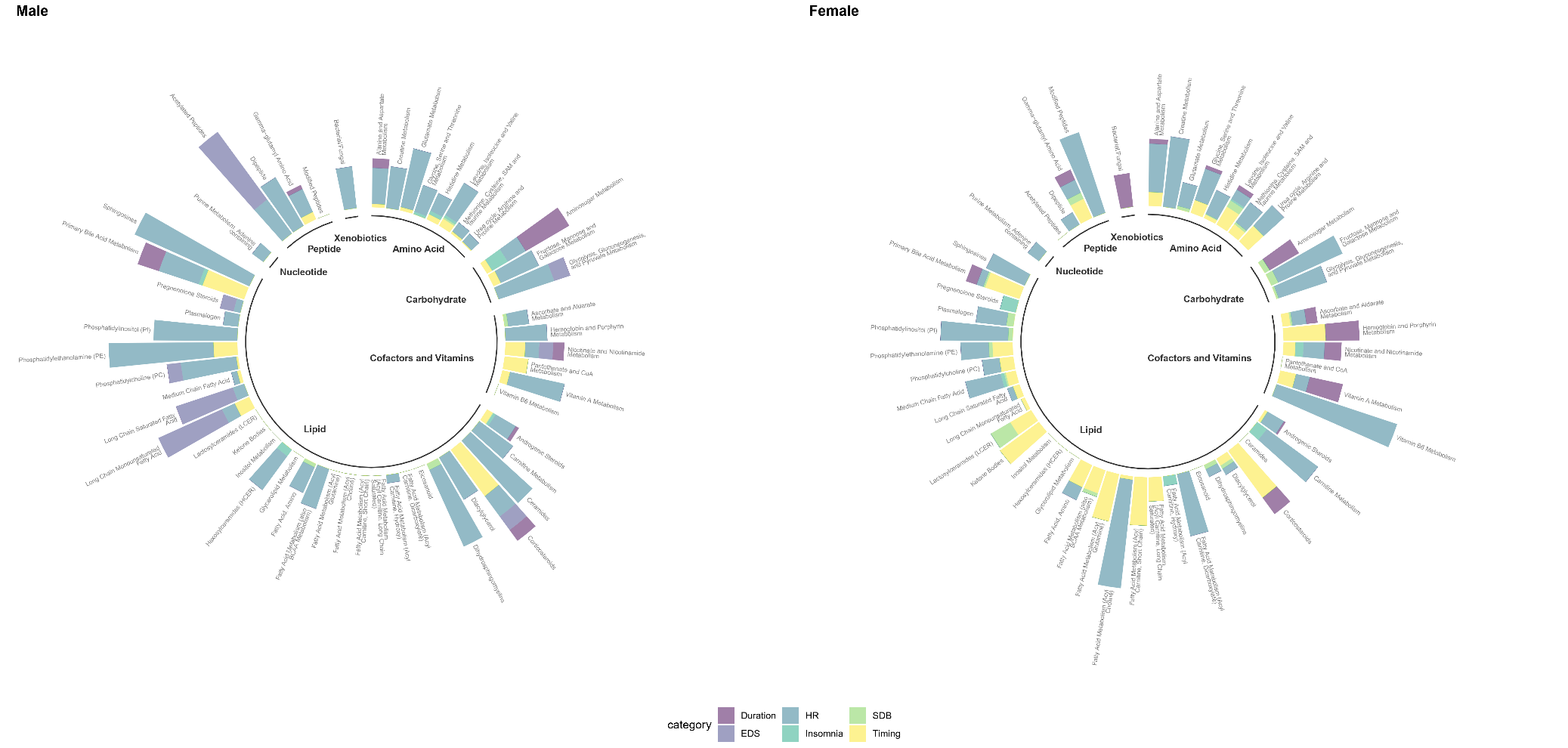


# Supplementary Figure S9. Network structural property metrics summarised by superpathway


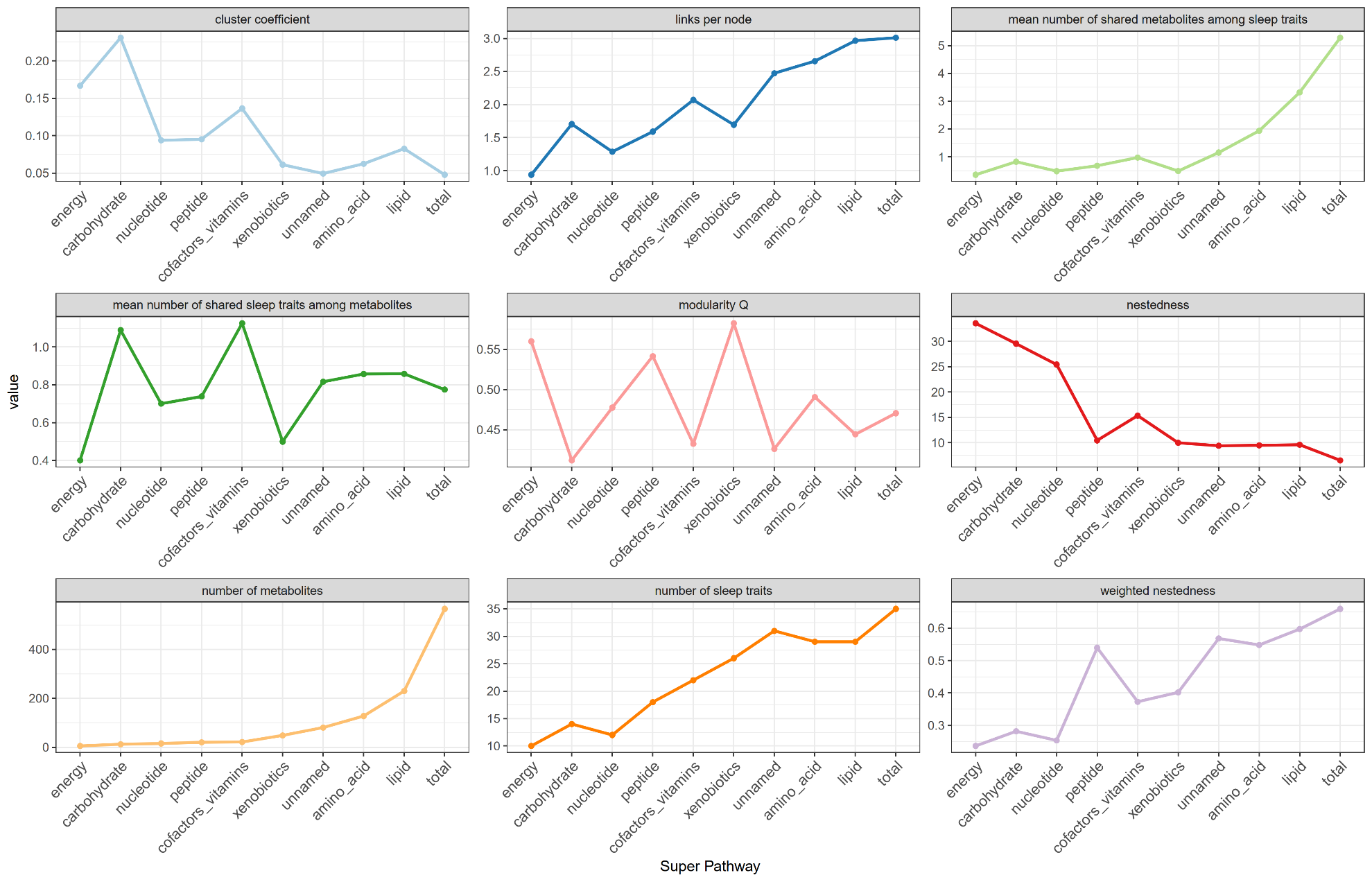


Network properties metrics were calculated for the bipartite network based on the significant associations between metabolites and sleep phenotypes (FDR-corrected *p*<0.05). Cluster coefficients is the number of realised links divided by the number of possible links [https://www.rdocumentation.org/packages/bipartite/versions/2.19/topics/networklevel]. Nestedness measures how the interactions of less connected nodes are a subset of the interactions of more connected nodes. A value of 0 indicates high nestedness, while a value of 100 indicates “chaos”. Weighted nestedness considers interaction frequencies of the network, proposed by Galeano et al.[^2^](https://sciwheel.com/work/citation?ids=186947&pre=&suf=&sa=0&dbf=0). It ranges between 1 (perfect nestedness) and 0 (perfect chaos). Modularity Q is a measure to quantify how well a network can be partitioned into different groups of nodes such that nodes that belong to the same group are more likely to be connected than nodes that belong to different groups.

# References

[1.    Faquih T, van Smeden M, Luo J, et al. A workflow for missing values imputation of untargeted metabolomics data. *Metabolites*. 2020;10(12). doi:10.3390/metabo10120486](https://sciwheel.com/work/bibliography/15082067)

[2.    Galeano J, Pastor JM, Iriondo JM. Weighted-Interaction Nestedness Estimator (WINE): A new estimator to calculate over frequency matrices. *Environmental Modelling & Software*. 2009;24(11):1342-1346. doi:10.1016/j.envsoft.2009.05.014](https://sciwheel.com/work/bibliography/186947)
